# Metallic elements combine with herbal compounds upload in microneedles to promote wound healing: a review

**DOI:** 10.3389/fbioe.2023.1283771

**Published:** 2023-11-03

**Authors:** Xiao Tang, Li Li, Gehang You, Xinyi Li, Jian Kang

**Affiliations:** ^1^ Department of Proctology, Hospital of Chengdu University of Traditional Chinese Medicine, Chengdu, Sichuan, China; ^2^ School of Clinical Medicine, Chengdu University of Traditional Chinese Medicine, Chengdu, Sichuan, China

**Keywords:** wound, microneedle, metallic elements, herbal compounds, acoustic and photoactive properties, drug delivery

## Abstract

Wound healing is a dynamic and complex restorative process, and traditional dressings reduce their therapeutic effectiveness due to the accumulation of drugs in the cuticle. As a novel drug delivery system, microneedles (MNs) can overcome the defect and deliver drugs to the deeper layers of the skin. As the core of the microneedle system, loaded drugs exert a significant influence on the therapeutic efficacy of MNs. Metallic elements and herbal compounds have been widely used in wound treatment for their ability to accelerate the healing process. Metallic elements primarily serve as antimicrobial agents and facilitate the enhancement of cell proliferation. Whereas various herbal compounds act on different targets in the inflammatory, proliferative, and remodeling phases of wound healing. The interaction between the two drugs forms nanoparticles (NPs) and metal-organic frameworks (MOFs), reducing the toxicity of the metallic elements and increasing the therapeutic effect. This article summarizes recent trends in the development of MNs made of metallic elements and herbal compounds for wound healing, describes their advantages in wound treatment, and provides a reference for the development of future MNs.

## 1 Introduction

Wounds are characterized by a deficit in skin tissue and can result from physical injuries (such as burns and surgeries), exposure to chemical irritants, or biological agents (such as diseases and bites). Once a wound is formed, its recovery will undergo coagulation, inflammation, proliferation, and remodeling phases (Ogawa, 2019). Wound recovery typically takes 2–3 weeks and is a complex physiologic process involving multicellular, multi cytokine interactions (Chong et al., 2007; Wang et al., 2019). Any disruption in these phases can result in delayed healing. The microenvironment of these non-healing wounds often exists in an aberrant state, characterized by persistent infection, malfunctioning oxidative stress responses, sluggish cell proliferation and migration, or irregularities in cell apoptosis (Qiang et al., 2021; Raziyeva et al., 2021). Complications arising from negligent management of the wound can increase the mortality rate of the patients and reduce the quality of their survival (Zarchi et al., 2015). Refractory wounds have become a widespread concern in various medical specialties, including dermatology, burn care, endocrinology, and various surgical fields.

**FIGURE 1:**
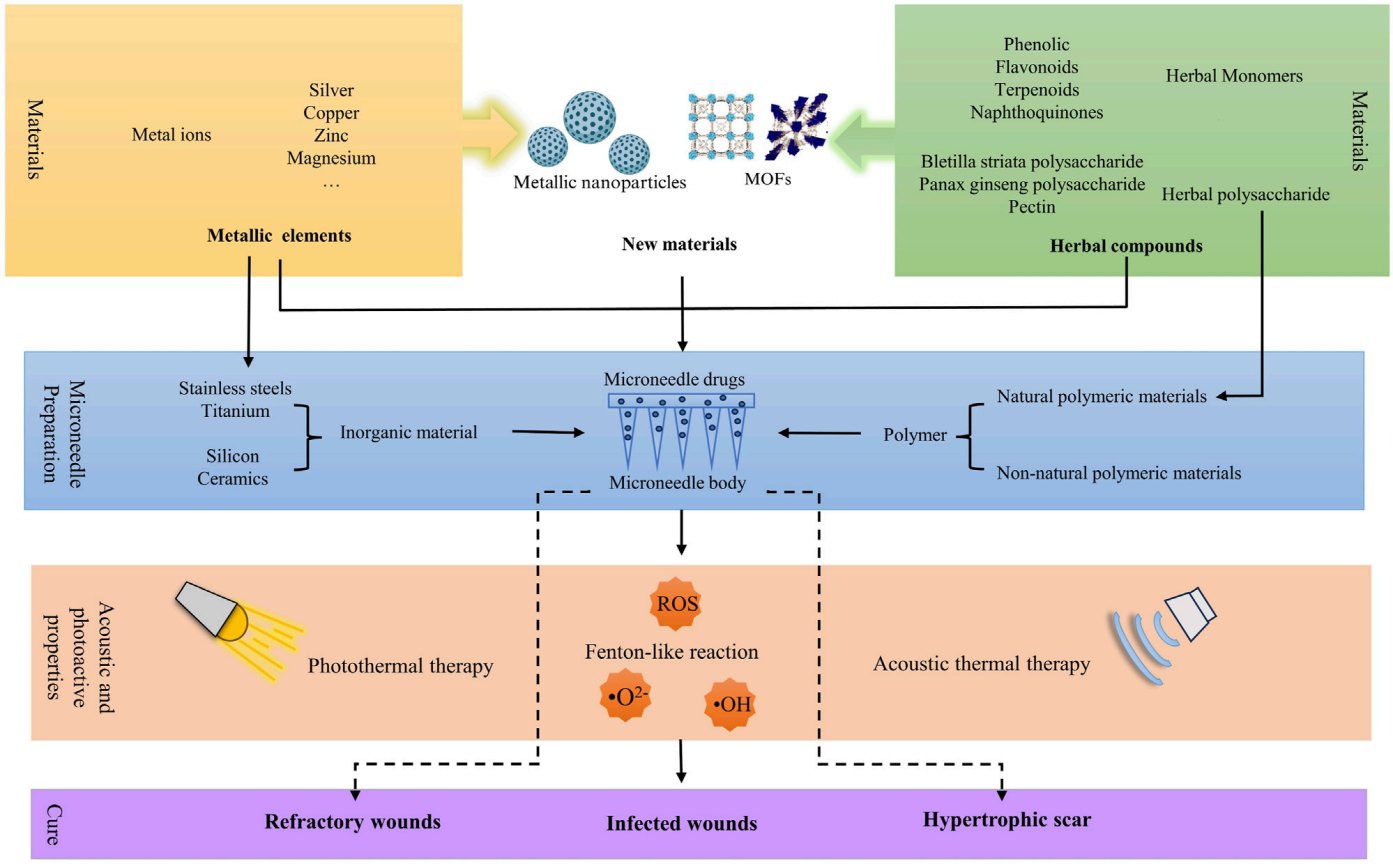
Manufacturing process and effects of MNs with metallic elements and herbal compounds.

The treatment of wounds with metallic drugs can traced back to the Hippocratic times. At that time, composite metals such as bronze were used to prevent wound infections (Krivoshapkin et al., 2014). However, due to defects in the purification process, highly concentrated and impure metal products can be skin irritants and can may even exacerbate injury zones (Ahn et al., 2019; Punjataewakupt et al., 2019). Fortunately, with the development of the pharmaceutical industry, purified metal ions have been recognized as good antimicrobial agents (Chen et al., 2022e). Modification of metal ions, metal nanoparticles (NPs), and metal-organic frameworks (MOFs) have also been further explored due to their low toxicity, controlled release, and sustained effects on the physiological functions of human cells (Alavi and Nokhodchi, 2020; Wang et al., 2023c; Tian et al., 2023). Another ancient remedy, herbal medicines, is abundant in bioactive components that can effectively intervene in various stages of wound recovery. They can expedite the resolution of infections, diminish inflammation, facilitate neovascularization, enhance skin cell proliferation, and even influence the apoptotic program during the remodeling phase (Liu et al., 2022a; Ning et al., 2022a; Li et al., 2023a). The development of monomers for the elaboration of their active ingredients has also reached a climax. Of greater interest is the fact that the interaction of the two drugs, metal, and herbal compounds, reduces metal toxicity, and the combination of the two exerts a greater efficacy, which is more suitable for the recovery process of the wound (Figure 1).

Currently, the two components have been widely added to hydrogels, foam dressings, electrospun, and other skin surface dressings for the treatment of refractory wounds (Khan et al., 2021; Jin et al., 2022a; Jin et al., 2022b). These topical dressings, particularly hydrogels with their porous structure, possess strong breathability and can maintain the wound’s moisture, effectively shielding it from harmful external environments. However, we have to face the fact that the drug delivery of these materials is limited to the wound surface, and the need to change the dressing several times to satisfy the therapeutic effect, which makes its use limited. The development of MNs addresses this gap by offering a novel and enhanced drug delivery approach. MN array penetrates the stratum corneum by piercing through the micrometer-sized dotted matrix needles, which not only deliver drugs to deeper layers of the skin but also expand the contact area of the drugs and localize them within specific cortical layers (Zhang et al., 2014; Amani et al., 2021). MNs made of porous materials can fulfill the need to load higher concentrations of metals and herbal compounds with highly biophilic activity. What is even more gratifying is that MNs can also sense wound pH, temperature, and light state, and provide intelligent controlled release of drugs according to wounds’ biological conditions (Chen et al., 2017; Guo et al., 2019; Shao et al., 2022).

The powerful, intelligent, and efficient drug-carrying capacity of MNs makes it necessary to select a drug that is most suitable for wound healing. The article summarizes previous reports on metallic elements and herbal drugs uploaded in MNs for wound healing. It delves deeply into the intricate mechanisms through which these two agents synergistically leverage MNs to effectively facilitate wound recovery. Furthermore, it systematically contrasts their therapeutic efficacies across diverse wound types and stages of healing, thus offering innovative insights that can shape the trajectory of future microneedle-based wound treatment systems.

## 2 Manufacture and therapeutic advantages of microneedles in wound healing

**FIGURE 2:**
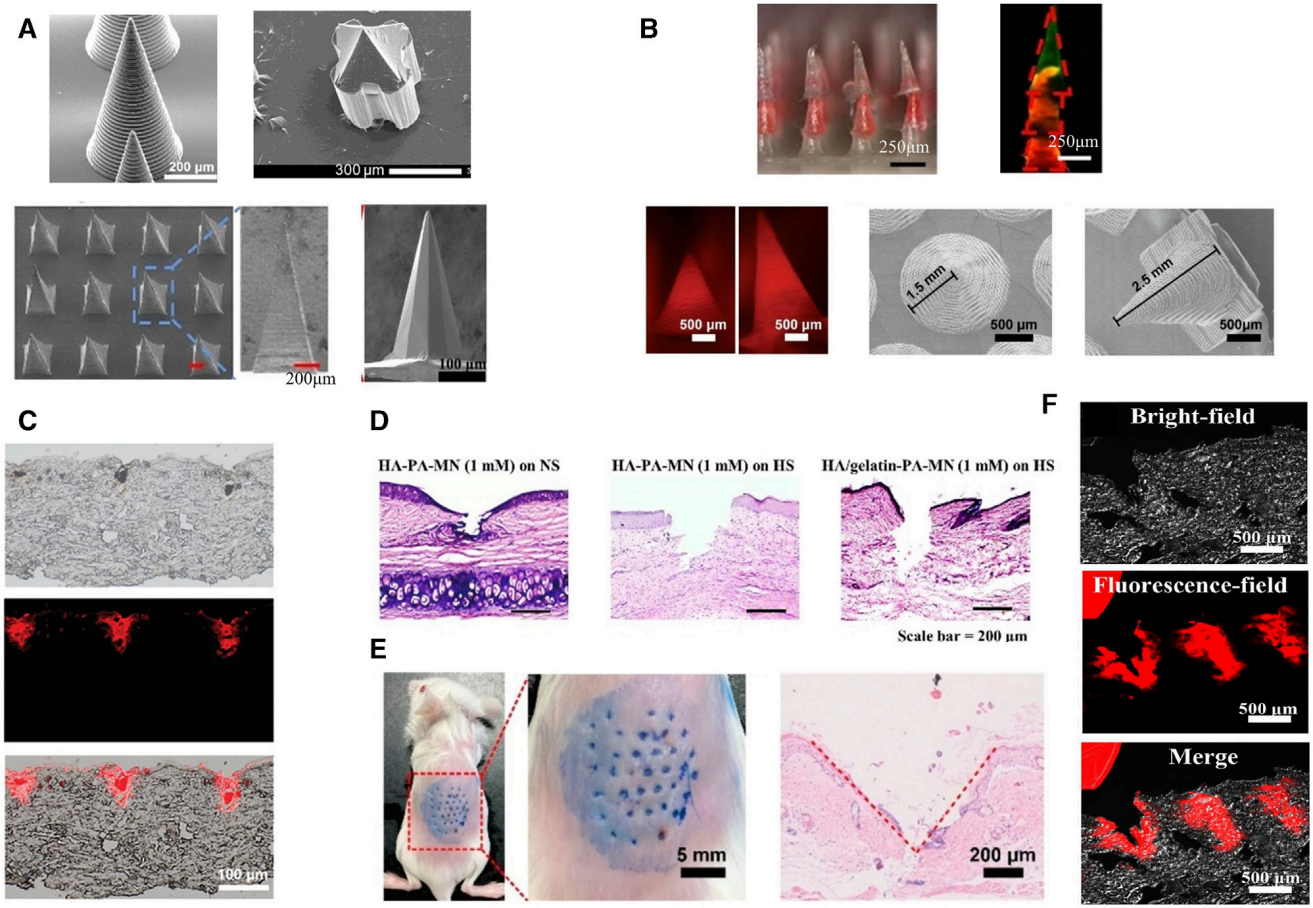
Microneedle morphology. **(A)** SEM images of MNs with different shapes (Wu et al., 2021; Yi et al., 2021; Hao et al., 2023; Zeng et al., 2023). Copyright 2023, Wiley. Copyright 2023, American Chemical Society. Copyright 2020, Wiley. Copyright 2023, Elsevier. **(B)** Configurations of various biomimetic microneedles (Deng et al., 2022; Liu et al., 2023b). Copyright 2023, Elsevier. Copyright 2022, American Chemical Society. **(C)** Histological images of cylindrical microneedles from **(A)** embedded in rat skin *ex vivo* (Zeng et al., 2023). Copyright 2023, Wiley. **(D)** Penetration ability of pyramid-shaped microneedles from **(A)** in rabbit skin (Hao et al., 2023). Copyright 2023, Elsevier. **(E)** *In vivo* skin penetration ability of lamprey teeth-like MNs (Deng et al., 2022). Copyright 2022, American Chemical Society. **(F)** Fluorescence images of histological sections of quill-like multilayer MNs after insertion (Liu et al., 2023b). Copyright 2023, Elsevier.

**FIGURE 3:**
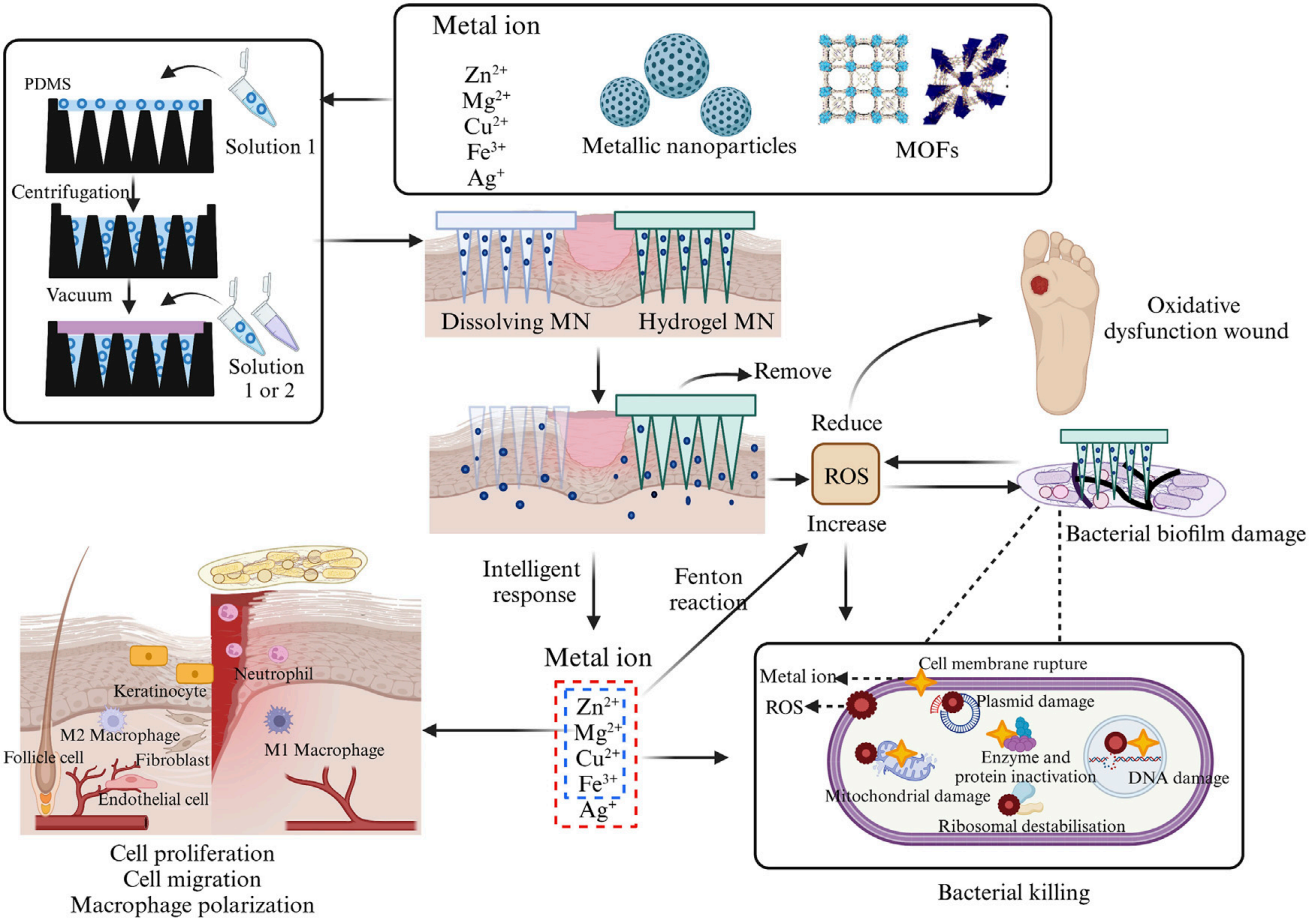
Incorporation of different configurations of metals into MNs fabrication process and their antibacterial and wound-healing processes. This picture was created by biorender.

Unlike the treatment of MN for other diseases, wounds have variable thicknesses at different healing stages and are covered by body fluids or bacterial membranes. Therefore, MNs in wound treatment need to be characterized by both strong penetration and high scratch force. Sufficient mechanical strength is the first essential to fulfill the cuticle penetration of MNs. Some MNs increase their needle length to increase their penetration depth. Others choose to modify the geometry of MNs. Previous studies have shown that three-sided pyramidal and four-sided pyramidal structures have better penetration (Tabriz et al., 2022) (Figure 2A; Supplementary Tables S1, S2). Regardless of how its physical form is altered, the mechanical strength of microneedles is primarily influenced by the properties of the materials used in their fabrication. As is known, microneedles are generally categorized into five types: solid microneedles, coating microneedle, hollow microneedle, dissolving microneedle, and hydrogel microneedle. The first three types of MNs are mostly made of high-strength structural materials, which are extremely strong and can easily penetrate the stratum corneum of the skin (Table 1). Their manufacturing process relies on laser cutting, laser ablation, photolithography, etching (dry etching and wet etching), three-dimensional printing, and micro stereolithography (Damiri et al., 2022). However, these MNs exhibit poor biocompatibility and fail to meet the demands of moist wound healing. While dissolving MNs and hydrogel MNs possessing high biophilic, high porosity, and intelligent response have emerged as the most extensively applied approaches in wound management (Gomes Neto et al., 2019; Chi et al., 2020; Yi et al., 2021; Haghniaz et al., 2023). Soft lithography utilizing polydimethylsiloxane (PDMS) is the most common process for the preparation of these two microneedles (Figure 3). Hydrogel MNs and dissolving MNs are formulated from a diverse range of materials (Table 1), yielding distinct mechanical strengths depending on the composition ratios. Selecting an appropriate material ratio becomes crucial to achieve the desired penetration of MNs into the stratum corneum. Consequently, considerable research efforts within the field have been dedicated to optimizing the material ratios for the two types of MNs. At the same time, to meet the need for secure fixation of MNs. The MNs were made into animal bionics MNs to form a certain angle between the skin, which makes up for the defects of weak gripping force (Figure 2B). Therefore, the manufacture of MNs for wounds requires mechanical strengths to better fit microneedles’ drug delivery properties (Figures 2C–F).

**TABLE 1:** Types of microneedles and constituent ingredients.

Type	Materials	References
Solid microneedle	Silicon; ceramic; stainless steel; titanium; glass	Al-Qallaf and Das (2009), Donnelly et al. (2009), Gittard et al. (2009), Li et al. (2017), Mugo et al. (2022)
Coating microneedle	Stainless steel; titanium; silicon; ceramics; poly (lactic acid) (PLA)	Peters et al. (2012), Vrdoljak et al. (2012), Boks et al. (2015), Zeng et al. (2017), Chinnadayyala et al. (2018)
Hollow microneedle	Silicon; PLA	Norman et al. (2013), Bolton et al. (2020)
Dissolving microneedle	Natural resourced	Vora et al. (2020), Zhu et al. (2020), Zhang et al. (2021d), Yang et al. (2022c), Loo et al. (2022)
	Chitosan (CS); hyaluronic acid (HA); Gelatin; silk Fibroin; herbal Polysaccharides; sodium alginate; maltose; pullulan polysaccharides	
	Unnatural resourced	Gao et al. (2021b), Permana et al. (2021), Deng et al. (2022), Wang et al. (2023b)
	Poly (vinylpyrrolidone) (PVP); poly (vinyl alcohol) (PVA); poly (lactic-co-glycolic acid) (PLGA); poly (ethylene glycol) diacrylate (PEGDA); gama-polyglutamic acid (γ-PGA); polycaprolactone (PCL)	
Hydrogel microneedle	HA; CS; PVA; poly (methyl vinyl ether-co-maleic acid); methacrylated hyaluronic acid (MeHA); gelatin methylacryloyl; polylactic acid	Demir et al. (2017), Skaria et al. (2019), Chi et al. (2020), Zheng et al. (2020)

Microneedles contain sufficient drug storage space and the tight adhesion to the cells gives it a highly efficient drug delivery and utilization rate. According to the delivery method, the MNs can be categorized as either externally dependent or needle-body dependent. When utilizing external drug delivery, MNs are mostly not therapeutically effective. Solid MN, hollow MN, or coating MN rely on the externally activated reservoir of adjunctive medication for drug delivery, with the MNs primarily serving as a “delivery conduit” (Mansoor et al., 2015; Tang et al., 2016; Jung et al., 2018). Another method of drug delivery adds drugs into the MN body during the manufacture, requiring microneedle deformation for drug release. For example, dissolving MN releases the loaded drug as the MNs melt. The swelling characteristic of hydrogel MN allows them to expand upon liquid absorption, yet they do not dissolve. The continuous channels formed within the gel network enable the drug to diffuse from the MNs into the skin along concentration gradients (Turner et al., 2021) (Figure 3). MNs can even be customized to monitor tissue interstitial fluids to achieve control drug release (Ullah et al., 2021; Shao et al., 2022; Zhao et al., 2023b).

Compared with the traditional subcutaneous injection, the rapid recovery of the MN administration site and the limited contact with nerve endings make MNs avoid severe tissue damage and pain during the treatment. And it is favored for its advantages such as transdermal delivery, high porosity rate, and good biocompatibility. However, it has to be recognized that rapid filling of defects is also necessary for wound healing. MN base can cover a part of the wound, but it does not fulfill the need to act as an extracellular matrix (ECM) and slows down cell migration. Therefore, MNs combined with other topical dressings such as hydrogels, and scaffolds have also been developed (Guo et al., 2022b; Ning et al., 2022b). These substances act as temporary fillers to isolate pathogens and provide a basis for the migration of surrounding cells. The porous structure also absorbs wound secretions and can carry a variety of nanomedicines thus promoting wound healing. In Table 2, we summarize the advantages and disadvantages of several topical dressings.

**TABLE 2:** Advantages and disadvantages of different wound dressing.

Type	Advantage	Disadvantage	References
Microneedle	Transdermal drug delivery	Residual sharps	Hardy et al. (2016), Donnelly and Larraňeta (2019), Shao et al. (2022), Jiang et al. (2023), Qi et al. (2023)
	Biodegradable	Physical irritation aggravates, inflammation	
	Intelligent drug release	Possible pain	
	High porosity rate		
	Wound monitoring		
	Excellent mechanical characteristics		
	Removed with minimal disruption of neoplastic tissue		
Hydrogel	Temporary ECM formation	Less cell attachment sites	Goodwin et al. (2016), Ghosal et al. (2017), Jin et al. (2021), Yuan et al. (2021), Yang et al. (2022a), Ryall et al. (2022b), Jiang et al. (2022), Muñoz-González et al. (2022)
	High porosity: strong water absorption and good air permeability	Limited to superficial cells	
	Biodegradable	Easy to remove	
	Easily removable	Inability to sustain drug delivery	
	Intelligent drug release	Uncontrolled drug diffusion	
Scaffold	Temporary ECM formation	Dissolution toxicity	Tallawi et al. (2015), Padil et al. (2020), Iliou et al. (2022)
	High porosity: strong water absorption and good air permeability		
	High flexibility		
	Excellent mechanical properties	Limited to superficial cells	
	Intelligent drug release	Fiber activity is unstable which can limit cell adhesion and proliferation	
Film	Flexible	Low permeability	Gopinath et al. (2004), Shi et al. (2020), Ryall et al. (2022b)
	Strong adhesion	Limited to superficial cells	
	Durable	Demolition destroys nascent tissue	
	Strong external barrier		

## 3 Application of metallic ion, metallic nanoparticle, and metal-organic frameworks in MNs

Currently, in addition to directly using metal ions to promote wound healing, various forms of metal elements, such as nanoparticles and MOFs, have also been widely applied. They accelerate the wound healing process by exerting functions like antimicrobial activity, promoting cell migration and proliferation (Figure 3). When metal drugs are added to MNs, their contact with the wound becomes closer, leading to greater therapeutic efficiency. This section will provide a detailed overview of the most reported types of metal elements used in MNs, aiming to gain a deeper understanding of the (Agarwal et al., 2019) advantages of metal microneedles in wound recovery (Supplementary Table S1).

### 3.1 Zinc

Zinc (Zn) exhibits excellent antimicrobial capabilities and contributes to human physiological functions, making it a significant contender to promote wound healing Inside bacteria, Zn disrupts the bacterial membrane potential, causing damage to the phospholipid bilayer structure, resulting in the formation of membrane pores, leakage of cellular contents, and ultimately cell death (Mishra et al., 2022). Once Zn^2+^ permeates into bacteria, it can bind to DNA phosphate groups, forming cooperative bonds, or react with thiol groups in bacterial proteins, inducing conformational changes and deactivation of proteins, thereby compromising cellular activity (Murali et al., 2021; Tavares et al., 2021) In the realm of maintaining human physiological functions, Zn^2+^ can increase superoxide dismutase (SOD) activity and scavenge intracellular reactive oxygen species (ROS) (Chen et al., 2022d). Zn supplementation accelerates the growth, proliferation, and migration of vascular endothelial cells, fibroblasts, and keratinocytes (Lin et al., 2017; Raguvaran et al., 2017). Additionally, as a cofactor for various proteins, Zn^2+^ expedites wound healing by regulating the expression of extracellular proteins (Chen et al., 2021). Furthermore, it is essential to note that in immune responses, Zn^2+^ acts by reducing caspase-1 expression to inhibit the NF-κB pathway in mast cells or by suppressing the expression of cyclooxygenase-2 (COX-2) and p53 protein levels, among other mechanisms, to alleviate inflammatory reactions (Murali et al., 2021).

When Zn^2+^ are loaded into chitosan (CS)-MNs, the antibacterial rate of *E. coli* and *S. aureus* is close to 100% at 0.6% (wt/wt), and the antibacterial potency is positively correlated with the concentration of Zn^2+^. The author’s team believed that the excellent antimicrobial effect of Zn^2+^-MN not only relies on the antimicrobial activity of Zn^2+^ itself, but the use of MNs makes the drug contact area increase by 14%–23% after stabbing into the bacterial biofilm. Meanwhile, higher mechanical strength is achieved by the coordination bond between Zn^2+^ and the -NH_2_ group of CS which resulted in a 49.5% increase in the MN’s mechanical strength (Yi et al., 2021) (Supplementary Table S1). In the presence of sufficient oxygen, Zn^2+^ can accept electrons to generate hydrogen peroxide (H_2_O_2_). Simultaneously, the binding of Zn^2+^ to targeted bacteria results in the production of a significant amount of ROS, leading the wound site into a state of heightened oxidative stress. The generation of these ROS can subsequently trigger apoptotic cell death in the remaining bacteria (Figure 3). However, a malfunction of oxidative stress in the wound may impair normal cell growth, and Cerium^3+^/Cerium^4+^ can exert activities similar to those of superoxide dismutase (SOD) and catalase (CAT), which makes the heightened oxidative stress caused by Zn^2+^ weakened. Therefore, even in diabetic infected wounds, Zn^2+^, while maintaining good antimicrobial activity, also utilizes the reducing activity of Cerium^3+^/Cerium^4+^ to inhibit the expression of NF-κB to reduce the release of pro-inflammatory factors to attenuate the expression of M1, while accelerating the migration of fibroblasts and angiogenesis (Yang et al., 2023b).

The antibacterial activity of Zn^2+^ is reassuring, but the surfaces of metal nanoparticles contain numerous atoms and vacancies. This enables Zn to exhibit a strong electric field enhancement effect and heightened chemical reactivity, enhancing its capability for substance adsorption and catalytic reactions (Qin et al., 2022). ZnO is a common form of zinc NP that can be absorbed by the porous structure of hyaluronic acid (HA), giving ZnO better water solubility and a positively charged surface (Zhang et al., 2023a). When the concentration of ZnO NPs increased from 200 μg/mL to 500 μg/mL, the antibacterial rate against *S. aureus* doubled, and the antimicrobial rate against *E. coli* increased from 60.55% ± 8.23% to 66.76% ± 6.98%, and it was biocompatible with the human umbilical vein endothelial cell (HUVECs) culture at this concentration (Zhang et al., 2023a). *In vivo* study of *S. aureus* infected wounds in mice, ZnO exhibited a promising bactericidal effect against the bacteria (Deng et al., 2022). When Zinc is integrated with other metal particles, it exhibits the potential to treat more “specialized” wounds. For instance, the incorporation of Zn_2_GeO_4_:Cu^2+^ nanorods within HA-MNs can lead to the continuous generation of reactive oxygen species (ROS), effectively disrupting bacterial metabolism and compromising the integrity of *Methicillin-Resistant Staphylococcus Aureus* (MRSA) cell membranes (Gong et al., 2022).

**FIGURE 4:**
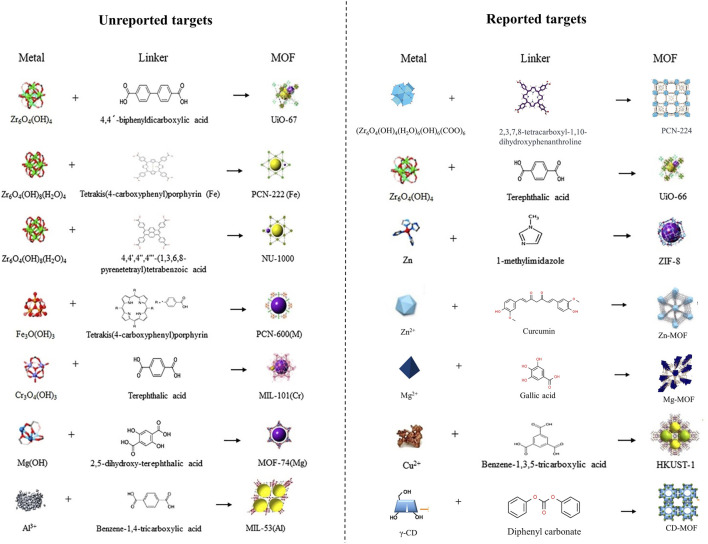
Preparation process of MOF or MOF-like materials added in wound-healing promotion MNs (Magri et al., 2021; Wu et al., 2021; Yin et al., 2021; Yang et al., 2023f; Zeng et al., 2023). Copyright 2021, Elsevier. Copyright 2023, Wiley. Copyright 2021, American Chemical Society. Copyright 2021, American Chemical Society.

The MOF is a type of nanoparticle with an inherent rigid structure. MOFs have a periodic network structure formed by the self-assembly of metal ions or metal atom cluster building blocks with organic ligands through coordination. Zn^2+^ combined with 1-methylimidazole (1-Melm) formed ZIF-8 displays a zeolite-like molecular sieve similar topology of rhombus dodecahedron (Figure 4). Young’s modulus was close to 1 MPa after adding ZIF-8 to a 3% concentration of methacrylated hyaluronic acid (MeHA) (Yao et al., 2021). Upon the dissolution of MNs, ZIF-8 also dissolved, leading to a sustained and even release of Zn^2+^. Notably, the release concentration was notably higher under acidic conditions (pH = 6.5) compared to alkaline conditions (pH = 7.4) (Qin et al., 2023). *In vitro*, experiments demonstrated that ZIF-8 tended to produce higher concentrations of ROS which mediated the death of *S. aureus*, and *P. aeruginosa* (Yao et al., 2021; Qin et al., 2023). Even when solely in contact with the wound site through an external dressing (hydrogel), ZIF-8 can still effectively achieve its antimicrobial effects (Han et al., 2023). Furthermore, as expected, ZIF-8 extends its wound-healing-promoting effects into the proliferative phase through the gradual release of Zn^2+^. *In vivo* experiments proved that with the addition of ZIF-8 to MNs, ZIF-8-MN showed higher anti-inflammatory activity than MeHA-MNs, and the expression of IL-6 and M1 marker CD68 decreased significantly, while the expression of M2 macrophage markers CD163 and CD206 increased in *S. aureus* whole-layer infected wound tissues. As an important indicator of the condition of the wound during the proliferative phase, the expression of the vascular endothelial cell marker CD31 and the fibroblast marker α-SMA was elevated, and richer collagen deposition was shown in the ZIF-8-MN group in Masson’s trichrome images (Qin et al., 2023). Other promising MOF, such as ZnTCPP, have demonstrated the ability to eliminate 99.73% of *P. acnes* with the assistance of ultrasound. This paves the way for further advancements in MOFs (Xiang et al., 2023).

### 3.2 Silver

Silver (Ag) is one of the most widely reported metallic elements in trauma medicine due to its favorable antimicrobial activity. Previous studies have demonstrated that hydrogels (Liu et al., 2022b), scaffolds (Hassan et al., 2021), foam dressing (Aduba et al., 2016), and films (Lv and Jiang, 2022) made with silver ions have shown good antimicrobial activity. When Ag^+^ is added to the biphasic scaffolds along with the dissolving MNs to form a dual drug delivery system, it exhibits antimicrobial activity against mixed biofilms of MRSA and *P. aeruginosa in vitro* (Su et al., 2021). Microneedles puncture the bacterial biofilm allowing Ag^+^ to penetrate deeper into the infection site. While some may be concerned that MN penetration increases the risk of deeper tissue, its insertion depth is limited and skin defects caused by MNs will be closed within hours thus reducing the risk of infection.

Silver NPs, another form of silver, have been recognized for their ability to achieve long-lasting release and exert stronger antimicrobial effects, making the use of MNs even more advantageous (Wang and Sun, 2021; Ryan et al., 2022). When sponge MNs made of polyvinyl alcohol (PVA) coated with Ag NPs inserted into agar discs with *E. coli* for 24 h, the zone of inhibition reached 31.5 ± 1.4 mm, whereas the range of the Ag^+^ group was only 28.2 ± 0.4 mm (Chen et al., 2023a). Meanwhile, Ag NPs-MNs showed a nearly 100% clearance rate against *P. aeruginosa* and *S. aureus* and the bacterial biofilm in rats (Permana et al., 2021). The death of bacterial relies on the interference of Ag NPs with bacterial membrane permeability and DNA damage (Venkatesan et al., 2015; Permana et al., 2021). An in-depth study showed that Ag NPs made of tannic acid (TA) achieved 99.99% antifungal efficiency against MRSA when added to CS-MN. The biofilm became rough and porous when MNs inserted into the bacterial biofilm, and 97% of MRSA was still killed in this recalcitrant infection state (Yang et al., 2022c). AgNO_3_ and filipin proteins formed nanosilver by incubation in an incandescent bulb (40 W) for 24 h. Coating it in MNs made of poly (ethylene glycol) diacrylate showed strong antimicrobial activity against gram-negative bacteria *E. coli* and *P. aeruginosa* and Gram-positive bacteria *S. aureus* and *S. epidermidis* (Gao et al., 2021b).

It has to be recognized that the cytotoxicity of Ag^+^ by binding to various biomolecules in the cells may diminish the pro-healing effect of MNs (Beer et al., 2012). By modifying Ag^+^ and achieving more precise control over its release, Ag NPs have increased their biocompatibility by reducing their impact on normal tissues (Zhang et al., 2021b). Polyphenols can effectively transform Ag^+^ into metallic Ag◦, thus attenuating its intervention in the cellular physiological processes of normal cells (Permana et al., 2021). When TA was introduced to Ag NPs during their incubation with L929 cells, it resulted in reduced cell death. The ability of TA to scavenge DPPH radicals and ROS might also contribute to alleviating its toxicity (Yang et al., 2022c). Elimination of the toxic effects of polyphenols on metal toxicity will be highlighted in section 6.1.

### 3.3 Magnesium

Diabetes mellitus (DM) is a significant factor contributing to non-healing wounds, and the dysfunction of oxidative stress is a common phenomenon in diabetic wounds. ROS serves as a crucial marker for oxidative stress dysregulation, are not only associated with aberrant glucose metabolism but also produced by bacteria in diabetic wounds (La Sala et al., 2018; Zhao et al., 2020). Although ROS production can kill bacteria, excessive ROS can also damage normal cells. Therefore, while utilizing metal elements to remove wound pathogens, their activity should be modified to induce the restoration of normal redox reactions in the wound. The MNs containing MgH_2_ NPs could react with the upper layer of chitosan hydrogel dressing to produce H_2_ bubbles. The bubbles could promote the rapid disassociation of the MNs and immobilization in the deep skin. The disintegration of Mg^2+^ led to a reduction of *E. coli* and *S. aureus* colonies by 90.4% and 96.1%, respectively. It also induced polarization of M1 macrophages, resulting in decreased expression of IL-1β and IL-6, while promoting the production of M2 macrophages. H_2_ produced by MgH_2_, in turn, binds to ROS and accelerates angiogenesis, fibroblast aggregation, and collagen polymerization rates in diabetic mice (Wang et al., 2023b).

The therapeutic effects of dual-layered MNs, composed of Mg-MOF combined with graphene oxide (GO) encapsulating Ag^+^, for diabetic wound treatment, become even more intriguing. In this type of MN, with increasing Mg-MOF concentration, the inhibition rates of DPPH free radicals and ROS gradually rise, demonstrating remarkable antioxidant performance. Within this microneedle system, the primary function of GO/Ag^+^ is to inhibit the growth of *S. aureus*, *E. coli*, or *P. aeruginosa* which has limited capacity for ROS clearance. While the degradation of Mg-MOF enhances HUVECs migration. The concurrent release of Mg^2+^ and TA increases diabetic wound granulation tissue width and vascular density, achieving synergistic enhancement and promoting wound healing (Yin et al., 2021). Furthermore, although Mg^2+^ harbors abundant antibacterial properties, its inherent physiological activity supports wound healing through multifaceted mechanisms, including promoting angiogenesis, stimulating collagen production, and facilitating cell proliferation and migration (Cui et al., 2022; Yang et al., 2023a) (Figure 3)

### 3.4 Copper

Similar to other metal ions, the incorporation of copper (Cu) ions also exhibits excellent antibacterial efficacy. When solely employing PAM/PDA-MNs patches, the survival rates of *E. coli* and *S. aureus* are only reduced to 40.1% and 42.5%, respectively, whereas the survival rates decrease to 28.86% and 21.65%, respectively, when using PAM-PDA/Cu^2+^-MNs (Liu et al., 2023b). Liang and others developed a microneedle system based on CuO_2_ and TiO_2_ (Liang et al., 2023). They found that TiO_2_ did not contribute to the antibacterial effect against MRSA and *P. aeruginosa*, while CuO_2_ alone exhibited antibacterial activity due to its predominant peroxo groups (Liang et al., 2023). In the meanwhile, the incorporation of TiO_2_ enhances the production of Cu^2+^ and H_2_O_2_, consequently amplifying the antibacterial efficacy of the MNs. After inoculation with MRSA on mouse wounds, CuO_2_/TiO_2_-MNs were observed to decrease the levels of TNF-a and IL-6 at the wound site and accelerate collagen deposition, thus substantiating their feasibility *in vivo* experiments (Liang et al., 2023). As a classic form of Cu-MOF, HKUST-1 can be formed through the combination of Cu^2+^ clusters and 1,3,5-benzenetricarboxylic acid. With the advancement of synthesis techniques, the particle size of HKUST-1 has been controlled within 567 nm from its original micrometer scale (Wang et al., 2018; Cun et al., 2022) (Figure 4). When HKUST-1 is encapsulated by graphene oxide (GO) and modified with 4-pyridinemethaneamine, HKUST-1 demonstrates a slower and sustained release of Cu^2+^, which ensures antibacterial activity while exhibiting lower toxicity to 3T3 cells. When modified HKUST-1 uploaded in PEGDA-MNs dissociated Cu^2+^ and nitric oxide (NO) continuously act on diabetic wounds, leading to elevated levels of CD31 and a-SMA (a neovascularization marker). Furthermore, it showcases potent pro-healing and reparative effects on the stratum corneum and granulation tissue, as revealed in Hematoxylin-eosin (H&E) staining (Yao et al., 2022).

The Cu_2_MoS_4_ can serve as a metallic nanosheet and is capable of hosting Au NPs. In addition to the antimicrobial effect exerted by the Cu^2+^ originating from the compound, the Cu_2_MoS_4_ nanosheet can infiltrate MRSA through a nano-knife-like mechanism. In this context, Cu_2_MoS_4_ also exhibits catalase (CAT) -like properties, effectively neutralizing excess ROS (Yang et al., 2023d; Shan et al., 2023). As for Au NPs, they exhibit glucose oxidase (GOx) -like activity, leading to the generation of H_2_O_2_ and glucose consumption. The interaction between Cu_2_MoS_4_ and Au NPs establishes a CAT—GOx cycle, ensuring a state where the wound is capable of eradicating pathogens while also preventing excessive oxidative stress (Shan et al., 2023).

### 3.5 Other metal elements

Zn, Mg, Ag, and Cu are widely studied in MNs applied for wound healing. But with the development of detoxification for metal elements and exploration of their redox activities, a group of promising metals has also come into view. Zirconium (Zr) is the 40th element in the periodic table. Zr_6_-cluster can form novel MOFs with its ligand 4-(carboxyphenyl) porphyrin (H_2_TCPP), known as PCN-224 (Figure 4). MOFs, constructed through a lattice-like structure formed by metal nodes and organic ligands, provide MOFs with a larger specific surface area, pore volume, and smaller density (Chen and Li, 2016). PCN-224 exhibits excellent activity against *E. coli*, *S. aureus*, and *P. aeruginosa*. When it is incorporated into HA-MNs, even with a reduced dose of 1/10, meropenem retains its original antibacterial efficacy (Zeng et al., 2023). While another form of Zr-MOF, UiO-66, modified with MoS_2_ exhibited excellent antimicrobial capacity against MRSA (Liao et al., 2022).

Tannic acid and Fe^III^-formed NPs have an average diameter of 100 nm. The FE^III^TA NPs can interact electrostatically with the primary amino groups of the MNs matrix material - polylysine, enhancing their antimicrobial capability. MNs incorporating FE^III^TA NPs exhibited faster recovery in diabetic mice compared to MNs without this substance. Wound biopsies conducted at multiple time points, specifically 3, 7, and 14 days, demonstrated significant reductions in TNF-α and IL-6, confirming the robust anti-inflammatory effect of FE^III^TA NPs (Wang et al., 2023a). MNs augmented with FE_2_O_3_/polydopamine (PDA) exhibited a dose-dependent reduction in the count of *E. coli* and *S. aureus* colonies in culture dishes which also effectively inhibited the formation of their bacterial biofilm. CD86 and CD206 were separately expressed on M1 and M2 macrophages, and the proportions of CD206, and IL-10 were increased in the Fe-added group, while CD86, IL-6, and TNF-α expression was significantly decreased (Li et al., 2023c).

## 4 Boosting metallic microneedle efficiency by acoustic and photoactive properties

During the wound healing process, timely and rapid resolution of inflammation in the inflammatory phase is a key focus for promoting wound recovery. However, within this phase, the excessive proliferation of bacteria leads to uncontrollable and intensified inflammatory reactions. Certain metal ions possess acoustic or photoactive properties that enable them to generate sonodynamic therapy (SDT), acoustic thermal therapy (ATT), or photodynamic therapy (PDT), and photothermal therapy (PTT). These effects can play a crucial auxiliary role in weakening bacterial growth at the wound site. This section aims to provide a detailed exploration of compounds possessing such activities when integrated into MNs, thereby enhancing the industry’s comprehension of the utilization of these substances.

### 4.1 Photothermal therapy

**FIGURE 5:**
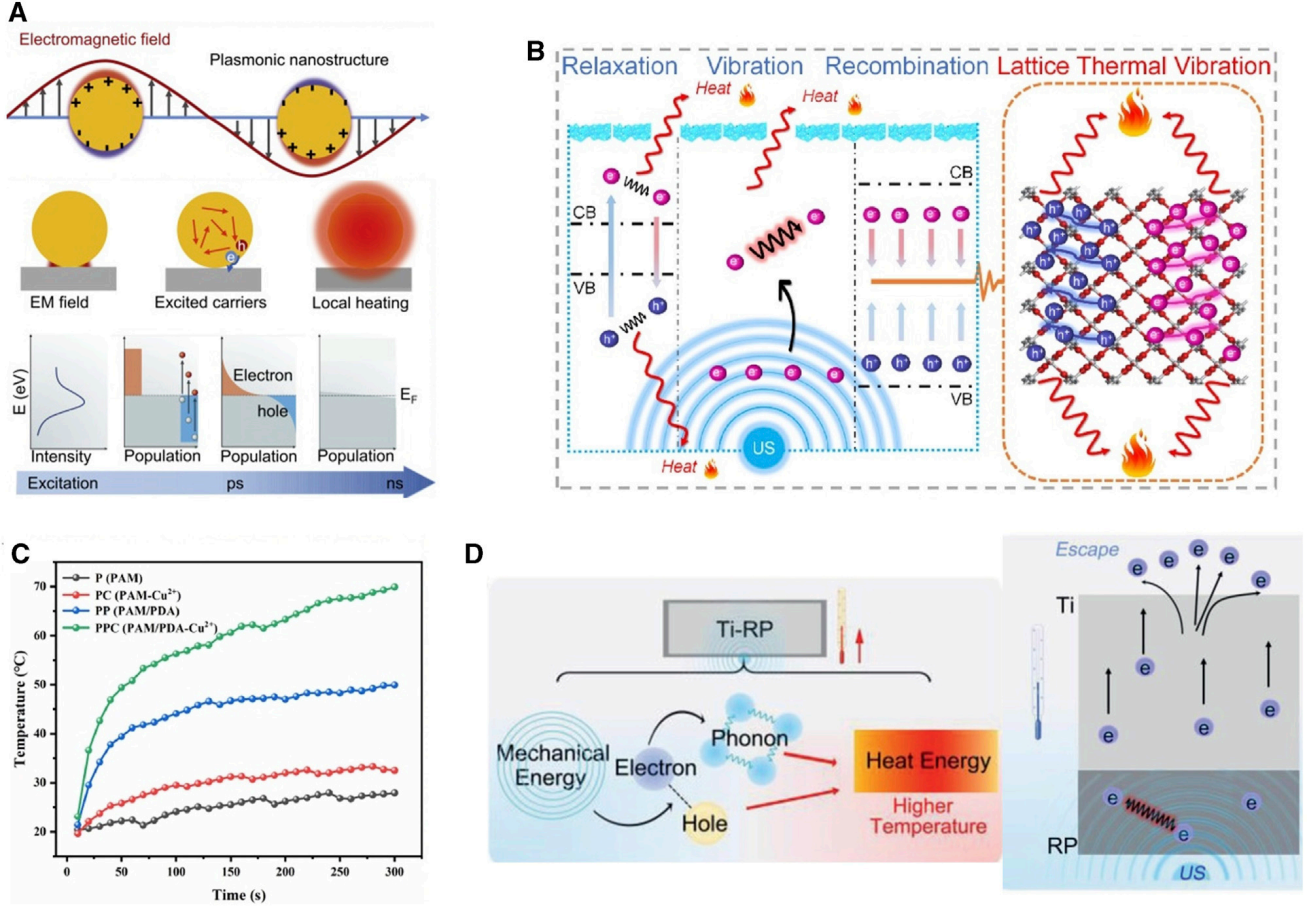
Properties of metal photothermal and sonothermal effects. **(A)** Light-induced collective oscillations of conduction electrons in metallic nanostructures and relaxation processes in surface plasma (Zhan et al., 2020). Copyright 2020, Elsevier. **(B)** Time-temperature curve for Cu-containing MNs under NIR irradiation (Liu et al., 2023b). Copyright 2023, Elsevier. **(C)** Sonothermal mechanism of CuO_2_/TiO_2_ in ultrasound (Liang et al., 2023). Copyright 2022, Elsevier. **(D)** Schematic representation of the generation of acoustic-thermal activity in Ti and red phosphorus, and the process of directed movement of surface electrons under ultrasound (Guan et al., 2021). Copyright 2020, Wiley.

Photothermal Therapy (PTT) is a commonly used approach that converts light energy into heat and utilizes this thermal energy for wound treatment (Sun and Hou, 2023). When ion NPs are exposed to light of specific wavelengths, the free electrons on the metal surface oscillate, activating surface plasmons (SPs) (Figure 5A). After SPs excitation, electrons within the metal particles undergo transitions, elevating the free electrons to higher energy states. At the same time, electrons accept the transfer of photon energy, causing lattice vibrations and generating heat. As electron-electron and electron-photon interactions tend toward equilibrium, SPs enter a relaxation process, which relies on the emission of photons through radiation or the creation of electron-hole pairs through non-radiative processes (Figure 5A) (Zhan et al., 2020; Xin et al., 2021). In order to achieve higher thermal energy conversion efficiency, photosensitizers need to exhibit a characteristic of a low band gap (Tao et al., 2019).

In MN systems, Cu and Fe can effectively generate SPR to fulfill PTT. The incorporation of Cu^2+^ into a complex formed through π-π interactions and hydrogen bonding with PDA and PAM meets the requirements for MN fabrication. Under 808 nm near-infrared (NIR) irradiation at a power density of 1.6 W/cm^2^ for 300 s, the surface temperature of MN patches can rise to around 50°C (Liu et al., 2023b). PDA itself serves as a photothermal material, and its temperature can increase under NIR illumination (Liu et al., 2014). However, research indicated that the interaction between PDA and Cu^2+^ forms a PDA/Cu^2+^ complex, which is crucial for enhanced photothermal performance. Therefore, in the PDA/PAM-MN group without the addition of Cu^2+^, the temperature of the MNs only increased by 28.5°C after 300 s of NIR irradiation (Xu et al., 2020; Liu et al., 2023b) (Figure 5B; Table 3). HKUST-1, as a carrier, can assist GO in exerting its photothermal effect. MNs formed by encapsulating HKUST-1 and GO can undergo repeated heating under NIR irradiation without deactivation (Yao et al., 2022). The photothermal performance of the entire material can be altered by adjusting the synthesis content of HKUST-1 raw materials (Fang et al., 2023a).

**TABLE 3:** Characteristics of acoustothermal and photothermal properties of metallic elements contain MNs.

Metallic elements	Activation condition	Duration (min)	Reach temperature (°C)	References
HKUST-1	Near-infrared light (NIR): 0.89 W/cm^2^	5	41.2	Yao et al. (2022)
Cu^2+^	NIR: 808 nm, 1.6 W/cm^2^	5	46.8	Liu et al. (2023b)
Cu_2_MoS_4_ + Au NP	NIR: 1064 nm, 0.6 W/cm^2^	5	56	Shan et al. (2023)
CuO_2_ + porous TiO_2_	Ultrasound (US): 1MHz, 1.0 W/cm^2^	5	CuO_2_: 45	Liang et al. (2023)
			TiO_2_: 52	
			CuO_2_+TiO_2_: 60	
Fe^III^/TA NP	NIR: 808 nm	10	55.2	Wang et al. (2023a)
Fe_2_O_3_	NIR: 660 nm, 1 W/cm^2^	5	46.8	Li et al. (2023c)
Anatase-brookite TiO_2_	US: 1 MHz, 1.5 W/cm^2^	15	41.8	Ouyang et al. (2023)
ZnO NP	NIR: 808 nm, 0.5 W/cm^2^	20	42	Zhang et al. (2023a)

In recent years, there has been a continuous increase in attention towards the porphyrin metal - Fe. The combination of Fe_2_O_3_ with PDA and GOx can form a Fe/PDA@GOx@HA-MNs. GOx and HA do not exhibit significant photothermal activity, when the concentration of Fe/PDA@GOx@HA increases from 2.5 μg/mL to 15 μg/mL, under the NIR illumination, the temperature rises from approximately 35°C to 46.8°C within 5 min (Li et al., 2023c) (Table 3). Additionally, TA contains abundant ortho-quinone and catechol groups. These phenolic groups coordinate with metal ions will form a metal-phenolic network, that can enhance its photothermal capacity (Zhang et al., 2021a). When Fe^III^ and TA combine, the Fe^III^ TA complex transforms the zeta potential of Fe^III^ from a positive value (+19.5 mV) to −21.6 mV under NIR irradiation. Additionally, its network exhibits a photothermal conversion efficiency of up to 77.3% (Liu et al., 2018). *In vitro* experiments demonstrated that when treated with NIR, increasing the concentration of Fe^III^TA NPs from 0% to 5% resulted in an enlargement of the inhibition zone diameter for *E. coli* from 1.83 ± 0.03 cm to 2.16 ± 0.06 cm, and for *S. aureus* from 1.34 ± 0.01 cm to 1.62 ± 0.035 cm (Wang et al., 2023a). It is worth noting that although PTT directly contributes to antimicrobial activity, photothermal also facilitates the dissolution of MNs. This allows the drugs to rapidly detach from the MN base, thereby enhancing the antimicrobial effect (Li et al., 2023c). Other MOFs formed by porphyrin metals like Zr (Zhao et al., 2022; Du et al., 2023) or metal elements with low electronic transition energy like Au (Chen et al., 2022c; Zhao et al., 2022), and Ag (Fan et al., 2018; Tong et al., 2020), have been loaded into topical dressings to display PTT. However, further validation is needed to assess the extent of enhancement within MNs.

### 4.2 Acoustic thermal therapy

Compared to light, ultrasound (US) has gained prominence due to its ability to penetrate deep tissues (Song et al., 2016). The US provides mechanical energy that can generate phonons through electron motion, resulting in lattice thermal vibration in the metal framework (Yu et al., 2019). Guan et al. found that in addition to electron vibration, relaxation, and recombination, phonons-directed transformation in the metal heterojunction also promotes ATT (Guan et al., 2021) (Figures 5C, D). When pristine TiO_2_ is made into oxygen vacancy-rich porous titanium oxide (OV-TiO_2_), the bandgap reduces from 3.24 eV to 2.16 eV, and narrowing bandgap will suppress the recombination of electron–hole pairs and enhance electron relaxation (Liang et al., 2023). OV-TiO_2_ enhances electron relaxation and electron-hole recombination performance, resulting in an acoustic-to-thermal conversion efficiency of 16% and generating a substantial amount of thermal energy. *In vivo*, experiments showed OV-TiO_2_-MNs have good antimicrobial efficacy in MRSA-infected wounds (Guan et al., 2021; Liang et al., 2023). Even in deep (bone) infection, the Ti-red phosphorus (RP) complex also successfully cures MRSA-infected rats by ATT (Guan et al., 2021). Among the various structures of Ti, anatase-brookite TiO_2_ (AB TiO_2_) has a better ultrasound catalytic mode than anatase TiO2, rutile TiO_2_, anatase-rutile TiO_2,_ and anatase-brookite-rutile TiO_2_ mode, which can achieve 41.8°C in 15 min to destroy the biofilm produced by bacteria. Because AB TiO_2_’s electron-hole recombination efficiency was lower than the others. Moreover, AB TiO_2_ promoted electron-hole separation better under the action of US, and AB TiO_2_ activation energy only requires 0.397 eV, making O_2_ on the AB more easily activated (Ouyang et al., 2023). Semiconductors such as Ag, Cr, Vanadium (V), and Bismuth (Bi) have been found to exhibit excellent ATT, but there are limited reports in MNs (Myronov et al., 2020; Meng et al., 2022; Zhao et al., 2023a; Yang et al., 2023e). Moreover, during combined microneedle therapy, it is essential to choose thermally controllable thermoacoustic materials to ensure the physiological activity of the surrounding tissues.

### 4.3 Acoustic and light energy supplementation enhances oxidative activity to promote wound healing

The role of sound and light is not limited to thermal activity. Metal ions can generate superoxide to kill bacteria through SDT and CDT. To understand the oxidation process, we first need to understand what is Fenton and Fenton-like reaction. Simply speaking, the Fenton reaction relies on the reaction of metal ions (mostly Fe^2+^) with H_2_O_2_ to produce highly reactive hydroxyl radicals (•OH), and peroxyl radicals (•O^2-^) (Wang et al., 2022a). The Fenton-like reaction, on the other hand, extends its reaction conditions to other metal ions (Cu^2+^, Ca^2+^, Zn^2+^) and oxidizing agents (e.g., peroxy groups) to produce peroxide ions (•OO-), hydroxyl radicals (•OH), etc (Tang et al., 2021b). Therefore, metal peroxides have been widely utilized because of the presence of both metal ions and peroxy groups (Wolanov et al., 2013; Shen et al., 2019; Bi et al., 2022). The introduction of external energy sources, such as light and sound, can be realized in the Fenton/Fenton-like reaction to supply energy and thus enhance the effect of the Fenton/Fenton-like reaction (Tang et al., 2021b; Jia et al., 2022).

**FIGURE 6:**
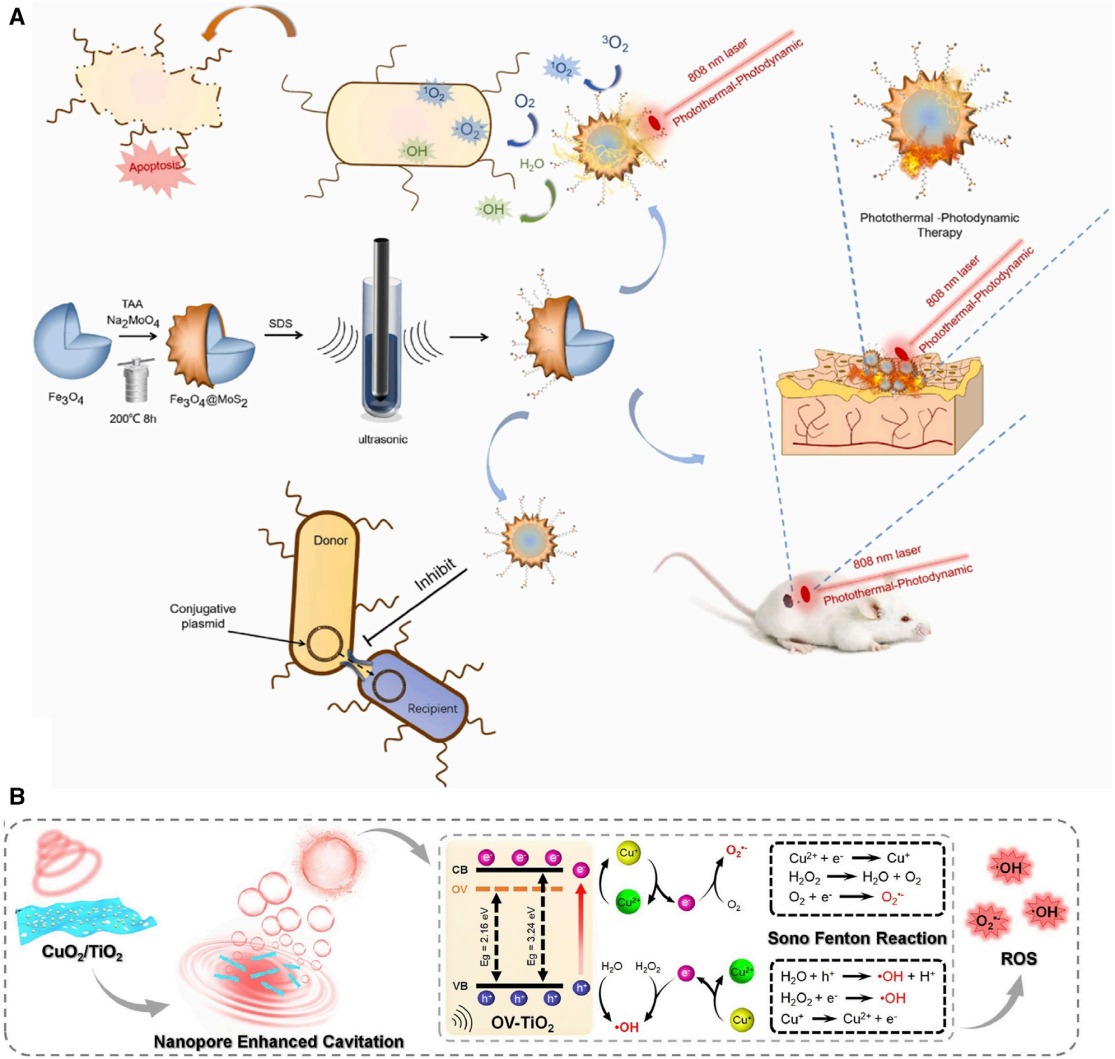
Fenton reactions of metal elements under light and ultrasound. **(A)** Therapeutic effects of Fenton reactions induced by Fe_3_O_4_ and MoS_2_ under illumination (Wang et al., 2022a). Copyright 2022, Elsevier. **(B)** Fenton reaction generated by CuO_2_/TiO_2_ under ultrasound (Liang et al., 2023). Copyright 2022, Elsevier.

Firstly, the Fenton/Fenton-like reaction of light achieves wound environment modification by activating photosensitizers while producing oxidants (Mosteo et al., 2020; Zhou et al., 2022). Metal ions containing porphyrin complexes have enhanced potential for generating ROS due to their photosensitivity and electron transfer capabilities. In the context of promoting wound healing, Fe_3_O_4_, when exposed to near-infrared (NIR) light, can absorb energy and convert O_2_ and H_2_O into abundant superoxide ions, demonstrating effective eradication of MRSA biofilms (Wang et al., 2022a) (Figure 6A). In addition, MOFs formed by porphyrin-like metal elements gradually release metal ions within the wound and these ions combined with oxygen anions (O^2^⁻) can release electrons that subsequently migrate to transition porphyrin elements to generate •OH(Deng et al., 2017; Zhang et al., 2021c). A typical porphyrin-like MOF, Zr-MOF, under NIR irradiation, converts H_2_O_2_ into ^1^O_2_, and the potential overflow of O_2_ may mitigate the harm caused by wound hypoxia (Chen et al., 2023c). Notably, Zr-MOF exhibits the highest reactivity in converting O_2_ into ^1^O_2_ within the first 10 min under irradiation and these superoxides are the key to rapidly eliminating bacteria (Zeng et al., 2023).

Similar to the light-activated Fenton reaction, the metal ions react with H_2_O_2_ to produce •O_2_- and •OH under the motivation of the US (Liang et al., 2023). Modified TiO_2_ (OV-TiO_2_) reduces the TiO_2_ bandgap, making electron transitions easier. Simultaneously, OV-TiO_2_ provides more adsorption sites for H_2_O_2_ and Cu^2+^ due to its enhanced adsorption ability. On the OV-TiO_2_ nanosheet, Cu^2+^ efficiently catalyzes the Fenton reaction, generating ROS (Figure 6B). The porous structure of TiO_2_ generates maximum, dense, and smaller O_2_ bubbles upon ultrasound exposure, which rupture to convert H_2_O molecules into •OH, accelerating bacterial eradication (Liang et al., 2023). Under US irradiation, the presence of TiO_2_ generates the highest levels of ROS, and at 1.0 W/cm^2^, *E. coli* survival rates were reduced to 2.6% (Osumi et al., 2017). Similarly, BaTiO_3_ formed through the compound of Ti, Ba, and O established a strong built-in electric field on the surface under US stimulation which could effectively catalyze the generation of ROS through piezoelectric separated electrons and holes on the opposite sides of BaTiO_3_ NPs (Liu et al., 2023a).

However, it must be acknowledged that excessive and prolonged generation of oxygen-free radicals may damage newborn cells thus prolonging wound healing time. Therefore, scientists have not only relied on Fenton reactions but also utilized materials such as metal ions (Au, Cr) (Chen et al., 2022a; Shan et al., 2023), natural compounds (Guo et al., 2022a), and self-extracted products (hierarchical microparticles) (Zhang et al., 2023a) to mitigate the harmful effects of excessive oxidation. It can be anticipated that as the development of metal particles advances further, the industry will undoubtedly create more sophisticated and efficient microneedle systems based on this therapy to promote faster and more effective wound healing in the future.

## 5 Therapeutic effects of herbal compounds microneedles in wound healing

Natural medicines, particularly herbal medicines, have a longstanding history of addressing skin injuries. Progress in drug purification techniques has breathed new life into the application of herbal medicines. New drug delivery methods make it necessary to gain a deeper understanding of the function of herbal compounds (Supplementary Table S2).

### 5.1 Herbal polysaccharide

Naturally sourced polysaccharides can be extracted from animals, plants, or human tissues, consisting of large molecular chains formed by glycosidic bonds which connect more than ten monosaccharide units (Yu et al., 2018; Otero et al., 2023). These natural products possess characteristics of low toxicity and high biological affinity. Among them, herbal polysaccharides (HPS) stand out due to their exceptional mechanical strength, enabling them to impart anti-inflammatory, analgesic, hemostatic, and anti-fibrotic properties (Yang et al., 2022b; Zhang et al., 2022b).

#### 5.1.1 Bletilla striata polysaccharide

The most widely reported addition to MN from HPS for wound healing was *Bletilla striata* polysaccharide (BSP). BSP showed the strongest sugar ring hydroxyl peak at 3407.75 cm^−1^ in the Fourier transform infrared (FT-IR). The backbone of BSP is mainly composed of (1→2)-linked α-d-mannopyranose and (1→4)-linked β-d-glucopyranose residues and glucose and mannose on the polysaccharide chain occur at a molar ratio of 3:1 (Wang et al., 2014; Hu et al., 2018) (Table 4). The primary requirement for MNs is to guarantee their biocompatibility. Utilizing a safe concentration of CS solution (4.0 wt%) mixed with BSP solution to build dissolving MNs exhibited a cell survival rate of 95% when co-cultured with L929 cells for 24 h (Yang et al., 2022c). A quantitative experiment demonstrated that BSP-MNs showed significant inhibitory effects on HSF proliferation, while normal fibroblast cell proliferation did not exhibit notable inhibition (Wu et al., 2021).

**TABLE 4:** Major herbal sources and structures of herbal compounds.

	Representative plants	Extraction site	Molecular formula	Chemical formula
Curcumin	*Curcuma longa; Curcuma aromatica; Curcuma zedoaria; Acorus calamus*	Stolon	C_21_H_20_O_6_	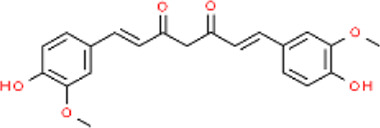
Protocatechuic aldehyde	*Ilex chinensis*	Leaf	C_7_H_6_O_3_	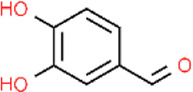
	*Salvia miltiorrhiza*	Roots		
Carvacrol	*Thymus vulgaris; Origanum vulgare; Satureja montana; Origanum majorana; Coriandrum sativum*	Whole plant	C_10_H_14_O	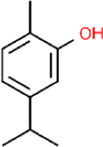
Tannic acid	*Quercus robur; Fagus sylvatica*	Bark	C_76_H_52_O_46_	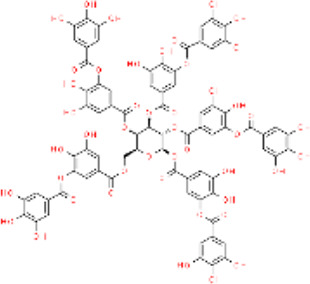
	*Vitis vinifera; Malus domestica*	Pericarp		
	*Vaccinium myrtillus; Ficus carica*	Fruit		
Catechin	*Camellia sinensis*	Leaf	C_15_H_14_O_6_	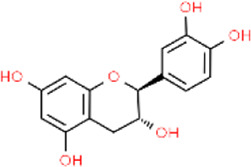
Gallic acid	*Terminalia chebula*	Whole plant/concentrated in fruit	C_7_H_6_O_5_	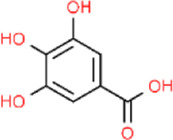
Quercetin	*Centella asiatica; Verbena officinalis; Camellia sinensis*	Leaf	C_15_H_10_O_7_ Ferenczyova et al. (2020)	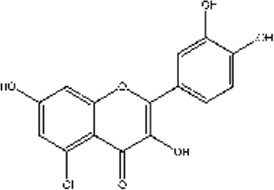
Luteolin	*Petroselinum crispum; Apium graveolens*	Stem	C_15_H_10_O_6_	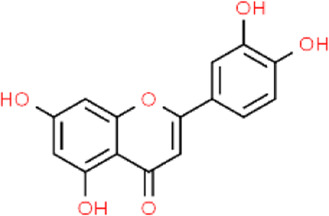
	*Gentiana lutea*	Root		
	*Perilla frutescens; Mentha aquatica; Leonurus japonicus; Verbena officinalis*	Leaf		
Asiatic acid	*Centella asiatica*	Whole plant/concentrated in root	C_30_H_48_O_5_	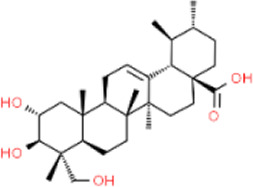
Asiaticoside	*Centella asiatica*	Whole plant/concentrated in root	C_48_H_78_O_19_	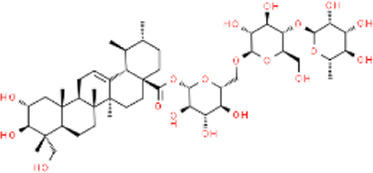
Tanshinone II_A_	*Salvia miltiorrhiza*	Root	C_19_H_18_O_3_	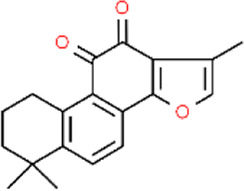
Shikonin	*Arnebia euchroma*	Leaf	C_16_H_16_O_5_	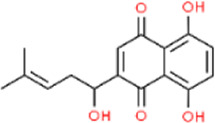
Bletilla striata polysaccharide	*Bletilla striata*	Stolon	Polysaccharide chain Qu et al. (2023)	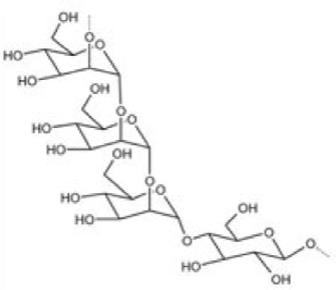
Panax notoginseng polysaccharide	*Panax notoginseng*	Stolon	Polysaccharide chain Wang et al. (2021a)	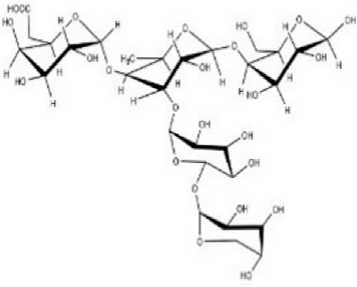
Pectin	*Premna microphylla*	Leaf	Polysaccharide chain Chi et al. (2021)	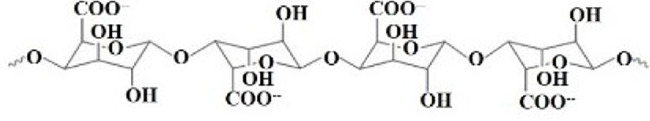

**FIGURE 7:**
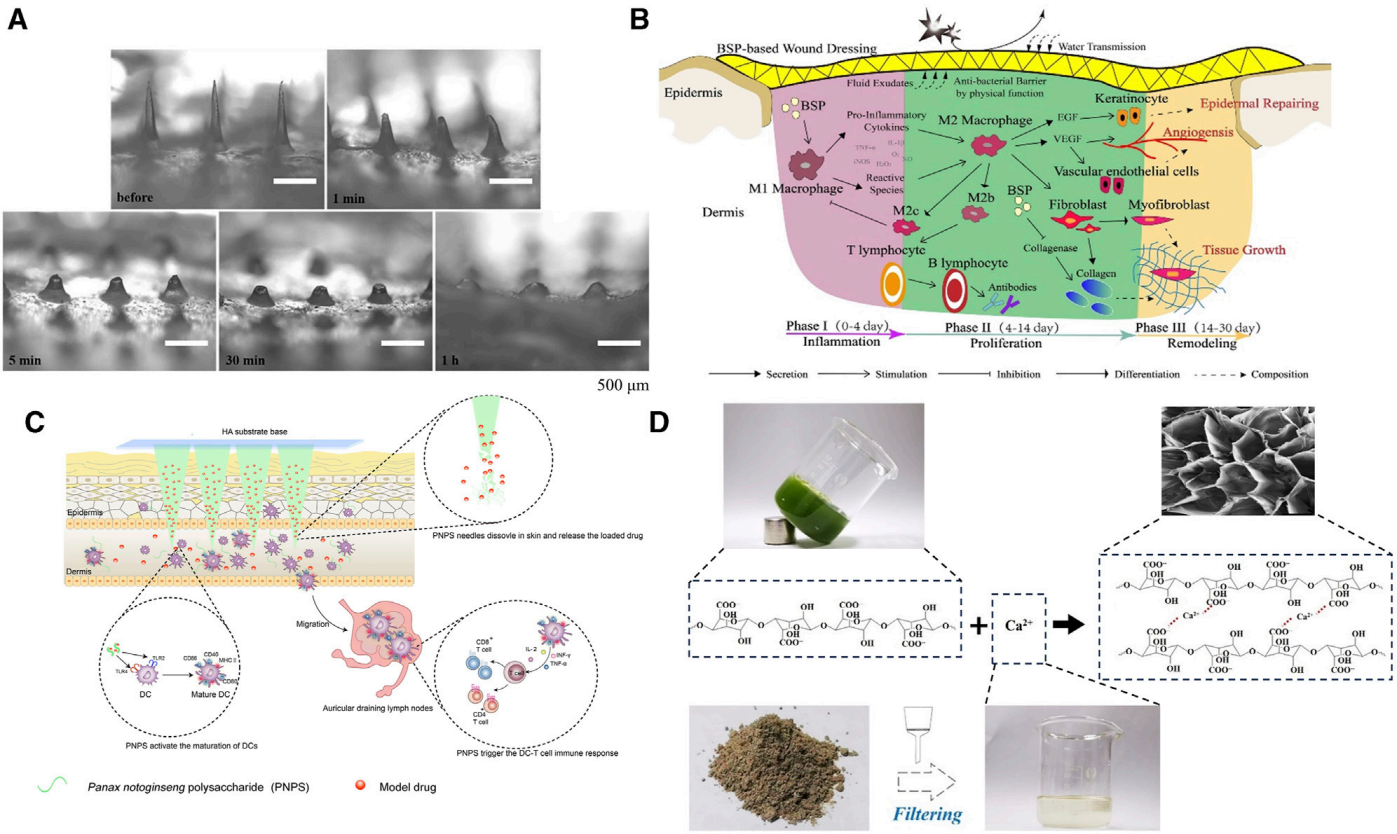
Mechanical properties and functional process of MNs made from herbal polysaccharides. **(A)** Dissolution of BSP-MNs after application onto rat skin *in vivo* (Hu et al., 2018). Copyright 2018, Elsevier. **(B)** Wound healing process mediated by BSP(Chen et al., 2018). Copyright 2018, Elsevier. **(C)** Post-implantation immune activation process of PNPS-MNs (Wang et al., 2021a). Copyright 2021, Elsevier. **(D)** Preparation process of pectin MNs derived from herbal sources (Chi et al., 2021).

It is believed that MNs achieving a mechanical force of 0.045 N/needle can effectively penetrate the dermis layer, higher rigidity strength can reduce the operation time, alleviate potential discomfort, and increase the likelihood of treating specific wound conditions, such as scar tissue or keloids (Gao et al., 2021a). BSP-MNs exhibit the shear thinning behavior of the pseudoplastic non-Newtonian fluid, facilitating the instantaneous penetration capability when the microneedle penetrates the skin. MNs made from pure 24% w/v BSP attain a mechanical hardness value of 0.52 N/needle, and at this concentration, the microneedles dissolve almost completely within 1 h without leaving any harmful residues (Hu et al., 2018) (Figure 7A). Another approach to enhance the mechanical strength of BSP-MNs is by incorporating additional long-chain polymers. Yang and others (Yang et al., 2022c) demonstrated that BSP and CS-formed MNs can establish strong molecular interactions within each other, leading to an impressive fracture force of 0.21 N/needle. When 15% w/v BSP is combined with carboxymethyl chitosan (CMCH), the MNs can withstand a force of up to 0.86 N/needle (Zhang et al., 2022a). Furthermore, BSP-MNs, often referred to as dissolving MN, also exhibit excellent drug delivery performance. After loading Rhodamine B (RB) onto BSP-MNs for 4 days, the cumulative permeation amounts for BSP-MNs loaded with 10 wt% and 5 wt% RB are 731.19 ± 75.52 μg and 389.95 ± 51.48 μg, respectively, whereas the quantitative release of RB from BSP skin patches is only approximately 10% and 5.82% of the concurrently loaded MN% (Hu et al., 2018).

The wound healing promotion of BSP is evident throughout the entire process (Figure 7B). During the hemostasis phase, the adhesion rates of BSP-MNs base to red blood cells and platelets reached 26.58% ± 6.42% and 29.61% ± 5.28%, which is comparable to another hemostatic agent, Yunnan Baiyao (BY)-MNs patches (27.82% ± 8.48%, 36.67% ± 7.60%) with both groups superior to the gauze group (3.47% ± 0.18%, 8.82% ± 1.89%). Prothrombin time (PT) is often used to measure extrinsic coagulation effectiveness. After the addition of BSP, the PT time is significantly reduced compared BY-MNs group (*p* < 0.01) (Yang et al., 2023c). Secondly, BSP demonstrates favorable anti-inflammatory and pro-angiogenic activities. When human gingival fibroblasts (hGFs) are co-cultured with lipopolysaccharide (LPS), BSP alone can reduce the content of TNF-α *in vitro* (Qu et al., 2023). As anticipated, histopathological examination of rats’ wounds confirmed that BSP containing MNs can decrease TNF-α content while increasing CD31 expression (Qu et al., 2023). Another experiment demonstrated that BSP exhibited a nearly fourfold increase in DPPH radical scavenging effect and ROS clearance activity compared to CS-MNs (Yang et al., 2022c). During the remodeling phase, CMCH/BSP-MNs can inhibit the expression of fibroblasts are recruited by transforming growth factor-β (TGF-β1), reduce fibroblasts’ expression of collagen, decrease the thickness of hypertrophic scars (HS), and reshape the arrangement of collagen fibers (Zhang et al., 2022a). Simultaneously, the viability of hypertrophic scar fibroblasts (HSFs), a key factor in HS formation, gradually diminishes, and qRT-PCR analysis demonstrates an elevation in BAX (a pro-apoptotic protein that eliminates mitochondrial membrane potential, activating the caspase cascade enzyme family and ultimately inducing cell apoptosis) expression by BSP-MN, suggesting a potential acceleration of apoptosis in HSF cells (Wu et al., 2021).

#### 5.1.2 *Panax ginseng* polysaccharide

When the concentration of PNPS-MNs solution reaches 10%, the mechanical strength of the MNs reaches approximately 0.16 N/needle (Supplementary Table S2). In comparison, PNPS-MNs exhibit weaker mechanical strength compared to other types of MNs. The PNPS-MNs with a height of 700 μm achieve a penetration depth of only about 200 μm in the dorsal skins of rats. However, its nearly 100% skin penetration efficiency ensures that drug delivery efficiency remains unaffected (Wang et al., 2021a). The primary role of PNPS on the skin surface is to promote acquired immunity. PNPS-MNs mediate the migration and maturation of dendritic cells (DCs). In the draining lymph nodes of mouse ears, the number of CD11c^+^/FTSC^+^ cells are 2.5 and 7 times respectively higher than that of dextran MN. Antigen-presenting cells, through enhanced antigen recognition, mediate an increase in the ratio of CD_4_^+^ and CD_8_^+^ T cells, leading to elevated local levels of TNF-α and INF-γ (Wang et al., 2021a) (Figure 7C). The abundance of galacturonic acid in PNPS enables it to target DCs by binding to the TLR4 receptor, subsequently inducing DC functional maturation through the TLR4/Myd88/NF-κB signaling pathway (Liu et al., 2020). Therefore, the potential immunostimulatory activity of PNPS in wound sites could exacerbate the inflammatory response. PNPS-MNs might achieve more promising results in non-healing wound caused by drug-resistant bacterial infections.

#### 5.1.3 *Premna microphylla* extract pectin

The reason why *Premna microphylla* can be formulated into a MN is due to its abundant content of pectin. However, the herbal juice requires the presence of a coagulant (Ca^2+^) to induce cross-linking of pectin molecules and initiate gel formation for microneedle preparation (Figure 7D). Additionally, the gel can maintain an optimal pore diameter when *P. microphylla* reaches a concentration of 6.67% and calcium-containing *Lolium perenne L.* ash reaches a 3.0% concentration (Chi et al., 2021). As a commonly found, environmentally friendly, and safe material, pectin is being increasingly explored for the development of MNs. When a composition of 15% w/w Poly (methyl vinyl ether-co-maleic acid) and 4% w/w pectin is used, the MNs transverse failure force is highest at different ratios (1% w/w 5%–2% w/w, 10% w/w–4% w/w, 10% w/w–2% w/w), reaching 0.25 ± 0.04 N/needle and the height reduction after axial fracture also reaches a peak of 27.60% ± 1.93% (Yilmaz et al., 2023). When pectin is combined with bovine serum albumin, the MNs have demonstrated excellent cell affinity and solubility (Demir and Kerimoglu, 2015). Currently, there is limited research on MNs produced from this product, and further exploration is needed for the manufacturing process of pectin microneedles. The incorporation of other materials (such as CS) may potentially offer a promising avenue to enhance their mechanical properties.

### 5.2 Phenolic compounds: curcumin, carvacrol, and protocatechuic aldehyde

**FIGURE 8:**
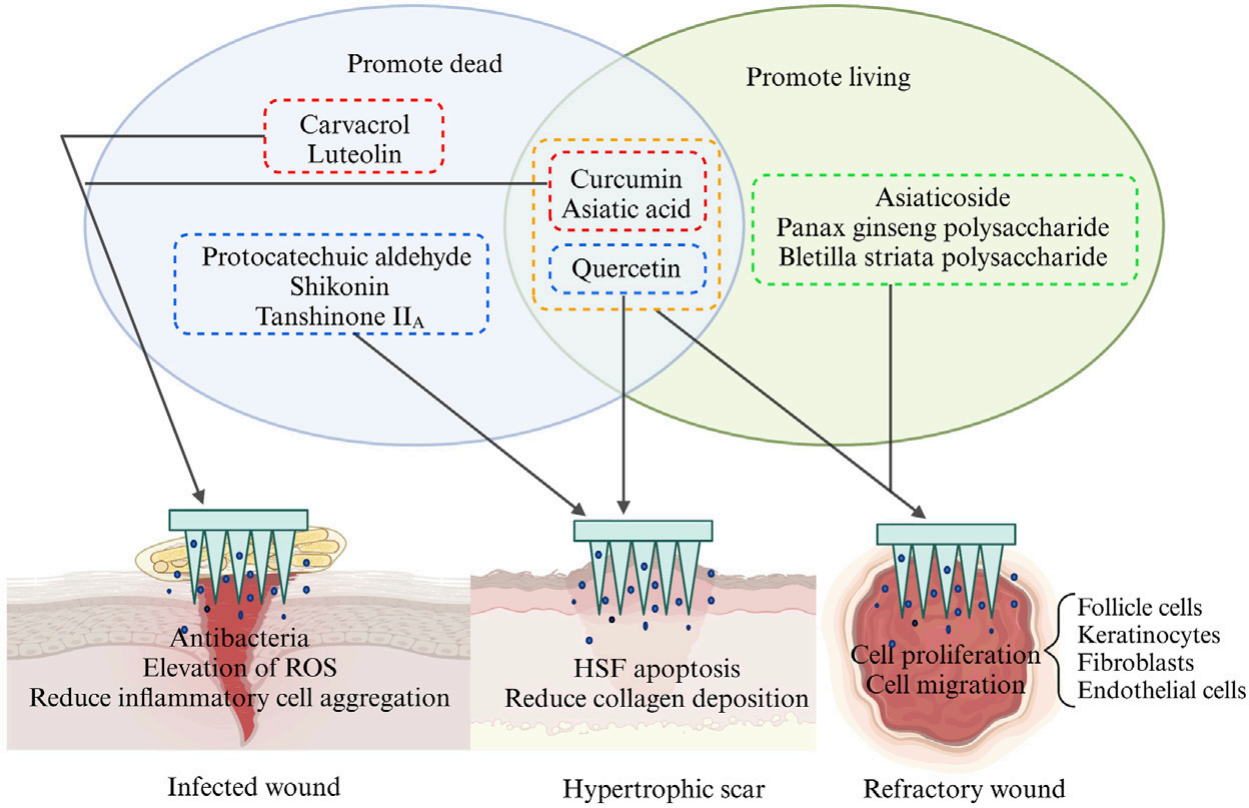
Schematic representation of the effects of different types of herbal compounds on different types of wounds. This picture was created by biorender.

Phenolic compounds are one of the most abundant categories in herbal medicine. They are organic compounds containing one or more hydroxyl groups and aromatic rings. These compounds can counteract oxidative stress and inhibit pro-inflammatory pathways, thereby promoting rapid wound healing (Cory et al., 2018; Chen et al., 2023d). Curcumin (CUR) is a type of polyphenolic compound found in various plants (Table 4). It is practically insoluble in water but readily soluble in lipids. The lipophilic nature of curcumin provides it with sufficient transmembrane permeability (Kumari et al., 2022). The role of curcumin in wound healing has been widely recognized. According to previous studies, curcumin can mitigate wound inflammation by blocking inflammatory signaling pathways and reducing oxidative stress. During the proliferative phase, curcumin promotes angiogenesis, fibroblast proliferation, and granulation tissue formation while also preventing excessive collagen deposition (Qu et al., 2018; Qian et al., 2022) (Figure 8). With the inherent advantages of MNs in drug delivery, the development of curcumin-loaded MNs is on the rise. When CUR-MNs are inserted into full-thickness pig skin, CUR-MNs completely dissolve within 45 min, and the release of curcumin reduces the accumulation of lymphocytes and neutrophils and reduces the inflammation of the wound (Shan et al., 2022). Mechanical strength testing demonstrated that the addition of CUR did not compromise the mechanical integrity of MNs even under a weight of 500 g. When curcumin reacted with Zn^2+^, a conical MOF was produced with an average nanoparticle size distribution of 424.9 ± 59.01 nm (Yang et al., 2023f). By day 15, the group treated with MOF-MNs nearly completely healed, and curcumin was positively regulated to promote M2 polarization in wound biopsies. Meanwhile, in the CUR-manufactured MOF group, there was an elevated expression of the fibroblast marker Ki67, as well as increased levels of CD31 (Yang et al., 2023f).

Carvacrol (CAR) is another phenol substance more widely found in nature. Using polycaprolactone (PCL) for encapsulating CAR, CAR/PCL NPs are formed with an average particle size of around 198 nm. The CAR/PCL NP-MNs exhibited an average penetration depth of 378 μm, accounting for 63% of the needle height (Mir et al., 2020). Due to the higher negative charge carried by CAR/PCL NPs, they exhibit stronger antimicrobial activity. Within 24 h, the cumulative release ratio of CAR NPs-MNs against both Gram-positive, Gram-negative bacteria, and MRSA with bacterial mortality >60%. In contrast, the antimicrobial activity of free CAR-MNs showed a noticeable decline (Kohli and Alpar, 2004; Mir et al., 2020).

**FIGURE 9:**
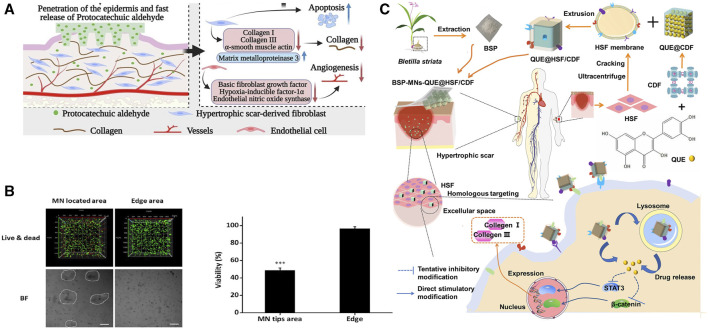
Effects of herbal MNs in anti-wound fibrosis. **(A)** Preparation of MNs containing BSP and quercetin and their anti-fibrotic pathways (Wu et al., 2021). Copyright 2021, American Chemical Society. **(B)** 3D local eradication effect of shikonin in HSFs culture (Ning et al., 2021). **(C)** Anti-fibrotic targets of original protocatechuic aldehyde (Hao et al., 2023). Copyright 2023, Elsevier.

The role of phenolics in wound repair is also reflected in the remodeling phase. Protocatechuic aldehyde (PA) possesses bioactivities such as inducing tumor cell apoptosis, reducing collagen synthesis, and inhibiting angiogenesis (Singh et al., 2018; Deng et al., 2020). Therefore, PA has been applied to inhibit HSFs viability. Compared to the control group, PA at concentrations of 50 μM, 100 μM, and 200 μM reduced HSF viability by 58.0% ± 2.4%, 74.6% ± 1.0%, and 88.6% ± 0.3%, respectively. PA can trigger apoptosis in HSFs, however, interestingly, at 50 μM and 100 μM, PA does not exhibit significant inhibitory effects on human normal fibroblasts. Further experiments demonstrated that PA mitigates excessive deposition of native proteins by suppressing the expression of COL1A1 (Collagen I), COL3A, ACTA1 (α-smooth muscle actin), and matrix metalloproteinase-3 (MMP3) in HSFs (Hao et al., 2023) (Figure 9A).

Phenolic compounds, such as tannic acid, gallic acid (GA), and catechins, play a role in promoting wound healing through their antioxidant activity and chelating with metal particles. We will delve into these aspects further in section 6.

### 5.3 Flavonoids

Flavonoids possess a fundamental structure comprising a benzene ring and a pyran ring interconnected via a C2-C3 unsaturated bond. They serve pivotal functions across diverse biological processes, encompassing anti-inflammatory, antimicrobial, antioxidant, and anti-cytotoxic activities (Singh et al., 2014) (Table 4). The most representative derivative of quercetin (QU) has been extensively studied and proven to promote good wound healing properties (Polerà et al., 2019). The utilization of QU is predominantly focused on the later stages of wound healing. In third-degree burn wounds, on the 10th and 24th days, the levels of neovascularization and CD31 in the QU-MNs group were higher than those in the Zn/Cu-MNs group. Zn/Cu-MNs primarily exhibited enhanced follicular cell migration capability, while the QU-MNs group primarily promoted follicular cell proliferation ability (Zhang et al., 2023b).

The limited water solubility of herbal compounds hinders their therapeutic efficacy. Encapsulation of hydrophobic substances before QU-MNs preparation can enhance QU’s bioavailability. In the process of QU-MNs fabrication, amphiphilic gelatin encapsulates QU to alleviate its hydrophobic nature (Chen et al., 2022f). The combination of QU/amphiphilic gelatin/gallic acid in MNs was found can decrease COL1A1, Col III and TGF-β1 levels in HS. The authors hypothesized that QU inhibits excessive collagen production by suppressing the TGF-β1/Smad signaling pathway and activating the PI3K/Akt signaling pathway (Chen et al., 2022f). Another technique involves the use of cross-linked cyclodextrin metal-organic framework (CDF) and HSF membrane to mitigate hydrophobicity and enhance cellular recognition. When MNs containing QU@HSF/CDF dissolve and are internalized by HSF, they inhibit STAT3/β-catenin signaling, thereby reducing collagen I/III production (Figure 9C). In this process, QU upregulates BAX and downregulates BCL-2 (an anti-apoptotic protein that maintains mitochondrial membrane potential and prevents the release of cytochrome c), suppressing the TGF-β/Smad pathway to induce HSF proliferation. Histological analysis of wound tissue also confirmed a significant reduction in collagen bundles in the MN group with QU addition. The QUE@HSF/CDF group exhibited comparable anti-fibrotic efficacy to the commercially available *Centella asiatica* triterpene cream. H&E and Masson staining revealed a decrease in fibroblasts and remodeling of collagen fibers, resulting in the skin at the treatment site closely resembling normal skin structure (Wu et al., 2021).

Killing bacteria to mitigate inflammation and inhibition is a key focus in shortening the inflammatory period of the wound. Nanomotors can form channels in bacterial biofilms, enhancing the penetration of antimicrobial agents within the biofilm and facilitating the diffusion of other drugs to the bacteria (Quan et al., 2019; Li et al., 2023b). As is widely recognized, luteolin (LE) exerts a potent effect in inhibiting bacterial growth (Guo Y. et al., 2020). After the incorporation of LE and nanomotors NPs (PMV/ICG/L-arginine) into MNs, the antibacterial activity of LE is significantly enhanced. In this scenario, the photosensitizer ICG can be activated by 808 nm NIR. The movement speed of nanomotor NPs increases from 1.95 μm/s to 5.60 μm/s, and the movement of negatively charged nanomotor NPs facilitates LE’s more efficient contact with bacteria. Nanomotor/LE-MNs achieve a wound healing rate of up to 90% within 7 days in rats (Chen et al., 2023b).

### 5.4 Terpenoids - *Centella Asiatica* extraction

Terpenoids are widespread phytochemicals, secondary metabolites with a variety of chemical structures, which can be categorized into semiterpenes, monoterpenes, sesquiterpenes, diterpenes, triterpenes, and tetraterpenes based on their chemical structures (Ghiulai et al., 2020). *Centella Asiatica* is widely used in Asia and contains abundant compounds such as Asiatic acid (AA) and asiaticoside (AS), both of which belong to triterpenoid compounds (Table 4) (Bylka et al., 2014). Ti_2_C_3_ MXenes are a novel type of single-layer nanosheets with an average thickness of 2nm, possessing a porous structure that provides a significant drug loading capacity exceeding 200% (Jastrzębska et al., 2017; Han et al., 2018). Loading AS onto MXenes does not increase its cytotoxicity, co-culturing HUVECs with AS at 5–30 μg/mL for 72 h results in higher cell viability. In diabetic mice, after the dissolution of AS-MNs, more vascular endothelial growth factor (VEGF) is generated through targeted binding to vascular endothelial cells. MXenes/AS-MNs exhibit a denser capillary density, with the highest expression of CD31 and Ki67 observed in immunofluorescence staining, followed by AS-MNs and MXenes-MNs (Wang P. et al., 2022).

As a derivative of AS, AA plays a dual role in antibacterial and wound healing promotion. The anti-bacterial activity is attributed to the membrane damage of bacteria caused by AA and the enhanced release of potassium ions and nucleotides sequentially. However, when the concentration of AA is increased to 0.5 μM it demonstrates optimal antibacterial efficiency against *E. coli* and *S. aureus* (Chi et al., 2021). *Premna microphylla* and AA-incorporated CHMN demonstrated remarkable wound healing function, yielding a regenerated granulation tissue of 0.96 ± 0.12 mm on the 9th day, a significant contrast to the control group’s measure of 0.35 ± 0.06 mm. The application of MNs optimized drug delivery efficiency, with the regenerated granulation tissue in the patch group measuring 0.86 ± 0.10 mm (Chi et al., 2021)*.* Cameron Ryall and others (Ryall et al., 2022a) devised two types of MNs to explore the optimal administration pathway for AS. Type 1 comprised a composition of 15% w/w PVA and 2% w/w CS, while type 2 was composed of 11% w/w PVP and 2% w/w CS. In the vertical fracture test, type1 MNs exhibited loading forces ranging from 1.26 N to 1.77 N, whereas type2 MNs showed forces of 0.43 N–0.51 N (Supplementary Table S2). By employing the mechanically stronger type 1 MNs, a remarkable 94% wound healing rate was achieved on the 14th day, in contrast to Tegaderm (a commercially available antimicrobial dressing) with a healing rate of only 62%. Tissue biopsy results revealed enhanced collagen deposition in the AA-MNs group, and the proliferation marker Ki67 of keratin-forming cells exhibited elevated levels in MNs loaded with AA, demonstrating a higher potential for tissue regeneration and healing (Ryall et al., 2022a).

### 5.5 Naphthoquinones

Naphthoquinones are naphthalene derivatives bearing two carbonyl oxygen atoms and are classified into three isoforms depending on the distribution of the carbonyl oxygen atoms (Qiu et al., 2018) (Table 4). Currently, subcutaneous hormone injections are most commonly used for the treatment of keloids and HS, but this treatment has a clinical recurrence rate of 9%–50%, and multiple injections may lead to hypopigmentation and skin atrophy (Ogawa, 2022). The unique structure of naphthoquinone imparts it with the ability to induce an increase in intracellular oxidative stress levels, leading to the effective treatment of scar hyperplasia via apoptosis (Coulidiati et al., 2015). Shikonin (SHI), an important herbal source of naphthoquinones, exhibited significant cytotoxicity against HSF when cultured with a TGF-β1 solution. In the presence of SHI, HSF showed minimal cell death within 24 h of exposure, with significant death occurring mainly after 48 h. Conversely, the SHI-MNs group observed a tangible HSF death in within 24 h, possibly due to the enhanced dissolution of shikonin facilitated by HA. 3D live/dead staining images revealed that HSFs embedded in a 3.1% agarose gel and in contact with the MNs exhibited significantly higher cell death signals. In contrast, HSFs in the non-contacted wound edge region exhibited higher survival rates, confirming the potent apoptotic effect of SHI on HSFs (Ning et al., 2021) (Figure 9B).

Tanshinone II_A_ (TSA) is also considered a member of the naphthoquinones family because of its typical 1,2-naphthoquinone structure (Qiu et al., 2018; Guo et al., 2020a). A mixture of TSA and PVP/CS was used to create MNs, which exhibited a slow and steady drug release of 55.68% within 24 h. The inhibitory effect of TSA on HSF showed a dose-dependent relationship at doses ranging from 0.2 μg/mL to 0.6 μg/mL. After 72 h of TSA treatment, HSFs in all groups exhibited reduced volume, became more rounded in shape, and showed a significant increase in cell death. Cells transitioned from an adherent state to a floating state, losing their characteristic morphology. In qRT-PCR analysis, high-dose (0.4 μg/mL) TSA-MNs treatment led to a more pronounced decrease in TGF-β1 compared to blank MN and free TSA groups. Low-dose (0.1 μg/mL) TSA and free TSA only increased Smad7 content in the experiment (Zhan et al., 2023).

The efficacy of herbal remedies in the treatment of skin injuries is well-established. However, in recent years, compounds extracted from edible plants have garnered widespread attention in the field of skin tissue regeneration due to their excellent biocompatibility, cost-effectiveness, ready availability, and high biological activity. Plants such as soybeans, tomatoes, and okra are rich in various antioxidant compounds that promote cellular migration and vascularization at wound sites, thereby facilitating wound remodeling (Xin et al., 2022; Fang et al., 2023b; Xin et al., 2023).

## 6 The interaction between herbal compounds and metallic elements within MNs

Metal elements have been criticized for their toxicity to skin cells. However, when metal ions interact with herbal compounds, a novel chemical structure is formed, reducing the potential cytotoxic effects of metals. The resulting new compounds, such as metal polyphenol networks (MPN), MOFs, and NPs, can combine with the matrix of manufactured MNs to achieve desired mechanical properties. Moreover, they can orderly dissolve in different wound environments without compromising their wound-healing properties. Herbal compounds, especially polyphenols, play a crucial role as mediators in reducing metal toxicity. In this section, we will elaborate on how metals interact with herbal compounds to achieve detoxification and enhancement, enabling MNs to exert superior wound-healing effects.

### 6.1 Herbal compounds bind to metal in advance to mitigate possible cytotoxicity

**FIGURE 10:**
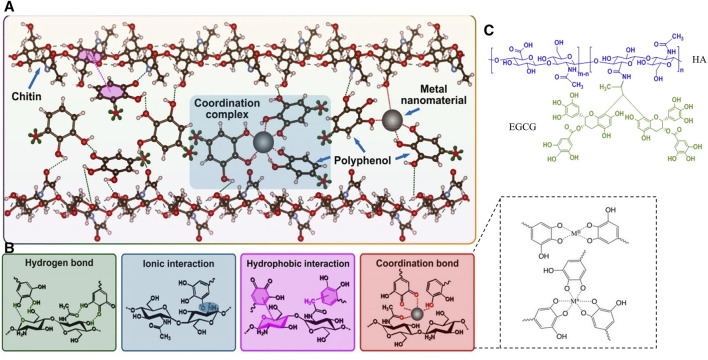
Metal-Phenolic Networks. **(A)** Network formed by chitin and metal-polyphenol interactions (Lin et al., 2022). Copyright 2022, Elsevier. **(B)** Connection pathways between metal, polyphenols, and chitin (Wei et al., 2018; Lin et al., 2022). Copyright 2022, Elsevier. Copyright 2018, Wiley. **(C)** Crosslinked structural compound formed between HA and EGCG (Liang et al., 2016). Copyright 2016, Elsevier.

Polyphenols are one of the most commonly used classes of metal detoxifiers in herbal extracts and can form stable complexes with metal ions through covalent interactions, thereby enhancing the bioavailability of metal ions. Phenolic hydroxyl (-OH) and carboxylic acid (-COOH) groups in phenolics contain lone pairs of electrons, which can form coordination bonds with the empty orbitals of metal ions. The polymerization process is influenced by factors including the type, charge, radius, and electron configuration of the metal ions, the structure and concentration of the phenolic compounds, and the reaction conditions (Rahim et al., 2016; Yun et al., 2018). This structure formed by polyphenols and metal ions is also known as metal polyphenol networks (MPN) (Figures 10A, B). Various metallic elements such as Fe, Cu, Zn, Ti, *etc.*, have been found to form a coordination bond with polyphenols and thus immobilize them in MPN, reducing the direct stimulatory effect of excessive metal ions on the traumatized cells (Lin et al., 2022). Within the microneedle system, polyphenolic compounds can undergo chemical conjugation with materials forming MNs, like HA, to create stable micellar nanocomplexes, capable of accommodating greater amounts of hydrophobic drugs (Liang et al., 2016) (Figure 10C).

In the metal-ligand coordination, the metal ions are viewed as Lewis acids which can accept electrons and the ligands are Lewis bases to donate electron pairs to metal ions. Metal ions are categorized into strong and weak acids based on their electron affinity, ionic charge state, and the size of metal atoms. They form ionic bonds (hard acids–hard bases), covalent bonds (soft acids and soft bases), and dynamic coordination bonds (hard acids–soft bases, soft acids–hard bases) with different bond strengths (Chen et al., 2022b). Therefore, the structural strength within MNs is closely related to the inherent properties of the metals and ligands involved. It is well-known that in the MN structures involving metal ions and -NH_2_, carboxyl, and phenolic groups, coordination bonds play a crucial role in cross-linking with polymer structures or other compounds compared to ionic and covalent bonds. Metal coordination bonds are advantageous due to their higher strength compared to other non-covalent bonds such as hydrogen bonds and π-π stacking and exhibit an overlapping effect with multiple coordination bonds (Luo et al., 2022). Ligands for metal coordination bonds can be provided by functional groups like -NH_2_, carboxyl, and phenolic groups. Compounds formed through the coordination between these ligands and metal ions are referred to as coordination complexes. The more coordination bonds formed between ligands and metal ions, the more stable the resulting chelate is. For instance, when 10 μM Fe^3+^ forms a robust bridging with two phenolic groups on catechol with an adhesion energy of 4.3 mJ/m^2^, while the bridging adhesion may be disrupted as the Fe^3+^ concentration increases (Yang et al., 2016) Similarly, the coordination bonds are formed through the interaction of lone pairs of electrons in -NH_2_ and phenolic groups with the positive charges on metal ions, resulting in electrostatic attractions. However, it is worth noting that besides the properties of the ligands themselves and the metal particles, the efficiency of coordination bond formation is influenced by various other factors such as pH, temperature, and pressure (Yu et al., 2013; Kim et al., 2022). In the meanwhile, pyrogallol or catechol moieties act as hydrogen bond donors in TA and can interact with carbonyl groups in PVP via noncovalent interactions, resulting in a robust drug delivery platform (Guo et al., 2021).

Compared to the simple network structure of MPN, MOFs represent a highly ordered crystalline structure. Within the MNs, Mg^2+^ reacts with gallic acid in water at pH 12 to synthesize Mg-MOF, which further self-assembles into nanoparticles through non-covalent π-π stacking interactions. The 3D structure of Mg-MOF helps preserve the antioxidant properties of gallic acid. *In vitro* experiments with MOFs demonstrate the promotion of HUVEC migration activity, and animal experiments confirm its affinity (Yin et al., 2021). Another approach involving the combination of TA and (Zn(NO_3_)_2_)·6H_2_O forms Zn-MOF, which does not exhibit inhibitory effects on human fibroblasts (Lamei and Hasanzadeh, 2022). However, if the concentration of Zn-MOF exceeds 3 mg/mL, there is a noticeable decrease in the proportion of fibroblasts. This approach not only achieves a detoxification effect but also employs intelligent dissolution under different pH conditions, allowing for the gradual release of metal ions and herbal compounds.

Another way to reduce toxicity using herbal compound is to modify the metal ions into a NP form. Ag^+^ in sodium citrate undergoes a reduction reaction to form Ag NPs, which are not uniform in size, fluctuating from 10–65 nm. Small (<33 nm) Ag NPs can lead to apoptosis in normal cells, but TA can control the reaction conditions to compensate for the defects in the size and shape of the Ag NPs and stabilize the Ag NPs particle size at 33 nm to reduce the mortality of L929 cells (Orlowski et al., 2018). The Ag NPs made of TA still showed positive zeta potential and could adhere to negatively charged bacteria, and the slow and continuous release of Ag^+^ from the Ag NPs avoided the cytotoxicity (Yang et al., 2022c). Similarly, green tea (*Camellia sinensis*) extract contains phenolics such as epigallocatechin-3-gallate (EGCG), epigallocatechin, and epicatechin (Rolim et al., 2019; Flieger et al., 2021). Catechin utilizes its reductive properties to convert Ag^+^ into metallic Ag° through -OH groups. After extracting green tea essence using high-pressure extraction, mixing it with 0.1 mM concentration of AgNO_3_ in a 1:8 ratio produces spherical Ag NPs with a particle size ranging from 28.76 ± 3.12 nm–35.71 ± 5.23 nm. However, reducing the ratio to 1:4 does not significantly alter the antibacterial minimum inhibitory concentration (MIC) and minimum bactericidal concentration (MBC) values (Tamuly et al., 2013; Permana et al., 2021). A more in-depth experiment involved the reaction of various types of polyphenols (such as TA, GA, resveratrol, epicatechin gallate, and cyanidin) with Au core-coated Ag coating NPs to form polyphenol-modified NPs. Polyphenol-modified Au@Ag NPs exhibited minimal mitochondrial toxicity. However, excessive levels of TA (>170 μM) could induce cell apoptosis and generate higher levels of ROS, affecting wound healing *in vivo*. In a cross-comparison, TA and GA-modified Au@Ag NPs showed the fastest migration of keratinocyte cells, promoting epidermal cell migration (Orlowski et al., 2020).

### 6.2 Herbal compounds weaken the established toxic effects of metals

As is widely known, heavy metal compounds such as mercury (Hg), arsenic (As), and lead (Pb) exhibit excellent antimicrobial activity by inducing oxidative stress and disrupting ion balance. However, due to the unique physiology of wounds, these heavy metal elements can rapidly and efficiently transported to the liver, kidneys, and brain tissues through damaged tissue, resulting in damage to normal cells. Once inside liver and kidney tissues, As and Hg upregulate uptake transporters (AQP9 and OAT1) and downregulate efflux transporters (P-gp, MRP2, and MRP4), leading to their accumulation (Wang et al., 2021b). Excessive metal elements rapidly bind to enzymes with thiol (-SH) groups within cells and DNA, disrupting normal cell physiological functions, thereby exacerbating oxidative stress, inflammation, and cell apoptosis in these cells (Yang et al., 2020). Xie and others (Xie et al., 2021) found that a moderate dose (400 mg/kg) of *Lycium barbarum* Polysaccharide (LBP) could almost restore the superoxide dismutase (SOD) levels, which were reduced due to lead exposure, to normal levels. Moreover, LBP reduced oxidative stress levels in renal tubular cells by downregulating the expression of Casp-3 and Bax, thereby slowing down cell apoptosis (Xie et al., 2021). Another study pointed out that *Malus micromalus Makino* extract is rich in epicatechin, chlorogenic acid, and quinic acid. These polyphenolic compounds, through their action, inhibited the increase in protein kinase C-α, cytochrome C, and Casp-3 levels, thereby reducing lead-induced endoplasmic reticulum stress (Wang et al., 2020). However, the damage caused by heavy metals to the human body is long-lasting. Pb-induced damage to liver cells and disruption of hormone secretion can lead to a secondary impact on the body. The compound herbal formulation Xiao Chai Hu decoction (a traditional herbal complex decoction) can upregulate the expression of Cyp3a11, Cyp4a12a, and UGT1a1 in the liver, thereby blocking the accelerated lipid metabolism pathway of Pb and promoting its elimination (Jia et al., 2021).

In fact, preventing the intake of high-damage metal particles is far more critical than cellular repair after ingestion. Therefore, low-toxicity metallic elements with biocompatibility, such as Fe, Zn, Mg, Cu, aluminum (Al), etc., are favored in wound medicine. But it must be acknowledged that, like heavy metals, excessive concentrations of metal ions from the aforementioned metals can induce various forms of cellular damage and apoptosis, resulting in increased wound inflammation and oxidative stress responses (Song et al., 2019). According to previous reports, substances such as saponins [rhodioloside (Tang et al., 2021a), *Panax ginseng* saponin (Wang et al., 2022c)], polyphenols (Orlowski et al., 2018) have been shown to rescue different types of cell damage caused by metal ions. Xu’s team (Xu et al., 2010) pointed out that ginsenoside Rg1 can inhibit the upregulation of divalent metal transporter 1/iron responsive element, reducing the lowering of mitochondrial transmembrane potential in MES23.5 cells, thereby alleviating iron-induced oxidative stress. In osteoblasts, ginsenoside Rg1 also enhances glutathione peroxidase and SOD activity to mitigate AlCl_3_-induced toxicity (Zhu et al., 2016). Polyphenolic compounds, on the other hand, have oxygen-containing functional groups that can chelate with metal ions to block their damaging effects. Cu^2+^ and Zn^2+^ can form a primary chelation site through β-diketone bridging, achieved by the loss of an enolic proton of curcumin, thus reducing the toxicity of polyphenolic substances (Balasubramanian, 2016; Sarawi et al., 2021). Furthermore, as previously mentioned, MNs incorporating polysaccharide compounds from natural extracts offer relief from oxidative stress imbalance resulting from bacterial death caused by metal ions.

Additionally, novel metal compounds derived from herbal medicines, such as MOFs, NPs, and MFPs, can slowly degrade under different pH and temperature conditions. This allows for effective doses without strong physiological toxicity, thus inactivating pathogens and promoting wound healing. We have reasons to believe that herbal medicine is effective in mitigating the toxic effects of metal ions on wound healing.

## 7 Conclusion and future perspective

Wound healing is a natural physiological response to tissue injury, which is essentially a balance between death (bacteria, HSF apoptosis) and living (vascular endothelial cells, keratinocytes, and fibroblasts). As a reliable novel drug delivery method, MNs deliver drugs to deeper layers of skin tissue, possessing desirable wound dressing features such as high biocompatibility, biodegradability, intelligent drug release, and easy replaceability. This enables the wound microenvironment to achieve a balance between cell death and survival (Verma et al., 2023). At the core of MN development, drugs can be incorporated with varying structures and sizes. The addition of metal ions, metal NPs, MPN, and MOFs enhances both antimicrobial and wound healing activities. By harnessing the acoustic and photoactive properties, along with the oxidative reactions of metallic elements, the antimicrobial properties of microneedles can be significantly enhanced. In the meanwhile, herbal compounds, as another promising substance, not only alleviate metal toxicity but also play a more effective role in various stages of wound healing by promoting cell proliferation and programmed apoptosis of wound cells. The advancement of new pharmaceutical technologies has enhanced the solubility, stability, bioavailability, and biocompatibility of herbal compounds.

The interaction between metals and herbal compounds broadens the potential applications of MNs as an innovative drug delivery method in wound healing. In this context, the toxicity of metals is mitigated by herbal compounds, and metal iron degradation efficiency is controlled. Additionally, with the assistance of metals, the antimicrobial efficacy of herbal compounds is enhanced, compensating for their inherent limitations. This synergy between metals and herbal compounds also enhances microneedle rigidity, achieving controlled release efficiency on the wound site and resulting in superior wound healing properties for MNs. However, we have to face the fact that even though MNs can achieve intelligent responses for controlled release, the issue of metal toxicity on newborn cells has not been entirely resolved. Furthermore, natural bioactive substances still encounter challenges, such as a lack of target specificity, susceptibility to hepatic metabolism, and potential allergenicity. The use of new materials generated under acoustic or photoactive stimuli may also lead to local overheating on the wound site, potentially damaging cells and slowing down wound healing. Additionally, in the clinical translation of metal/herbal compound-loaded microneedles, it is still in the preclinical trial stage. Concerns surrounding microneedle quality control, cost-effectiveness, safety, and rapid, low-cost production continue to persist. However, it is undeniable that microneedles offer unique advantages, including intelligence, precision, and controllability, setting them apart from other wound dressings. It is foreseeable that MNs will play a significant role in clinical wound treatment and may open up new avenues for treating other medical conditions.
