# Supplementary material for: Metallic elements combine with herbal compounds upload in microneedles to promote wound healing: a review
Source: Front Bioeng Biotechnol. 2023 Nov 3;11:1283771. doi: 10.3389/fbioe.2023.1283771 (PMC10655017; doi:10.3389/fbioe.2023.1283771)
Supplement: Supplementary file 1 [file Table1.docx]

**Supplementary table 1:** Characteristics and therapeutic targets of microneedles made with metallic elements.

| Metal formation | Foundation | Other compounds | Characteristics | Mechanical performance | Wound types | Efficiency | Reference |
| --- | --- | --- | --- | --- | --- | --- | --- |
| Zn^2+^ | CS | None | Shape: octagonal pyramid  Hight: 430 μm  Width: 160 μm  Tip distance: 615 μm | 0.4% wt/wt Zn^2+^ mechanical strength: 0.626 N/needle | NA | Anti-*E. coli*/ *S. aureus*;  Weakening of bacterial biofilm | (Yi et al., 2021) |
| Zn^2+^ | HA | Sericin^3+^/Sericin^4+^ | Shape: conical  Hight: 600 μm  Width: 200 μm  Tip distance: 500 μm | Destructive force: 1.4 N/patch | MRSA infected diabetic wound | Anti-MRSA;  Produce ROS;  Increases angiogenesis | (Yang et al., 2023b) |
| ZIF-8 | MeHA | None | Shape: conical  Hight: 700 μm  Width: 310 μm  Tip distance: 500 μm | In vitro insertion depth: approximately 200 μm | *S. aureus* infected wounds | Anti-*E. coli*/ *S. aureus*;  Reduces inflammation;  Increases angiogenesis;  Collagen deposition | (Yao et al., 2021) |
| ZIF-8 | MeHA | Dimethyloxalylglycine | Shape: pyramid  Hight: 800 μm  Width: 300 μm | Mechanical strength: 0.07 N/needle;  In vivo: 100% penetration of rat dorsal skin | *S. aureus* infected wounds | Anti- *S. aureus*/*P. aeruginosa*;  Reduce oxidative stress;  Reduces inflammation | (Qin et al., 2023) |
| ZnO | MeHA | Hierarchical microparticle/VEGF/bFGF | 11×11 matrix  Shape: pyramid  Hight: 800 μm  Width: 300 μm  Tip distance: 600 μm | Compressive strength: 2.1 N/ patch | Diabetic wound | Anti-*E. coli*/ *S. aureus*;  Scavenges ROS;  Promote angiogenesis; Promotes collagen deposition | (Zhang et al., 2023a) |
| ZnO | PVA | Sericin | Lamprey teeth-like microneedle with 19 short needles in the center and 16 long needles at the edge.  Hight of the long needle: 2500 μm  Hight of the long needle: 1500 μm | Failure force: 1.2 N/patch | *S. aureus* infected wounds | Anti- *S. aureus*/ *E. coli*;  Promote collagen deposition;  Promotes angiogenesis;  Promotes hair follicle generation | (Deng et al., 2022) |
| Ag NP | CS + Bletilla striata polysaccharide (BSP) | Tannic acid | 20×20 matrix  Shape: pyramid  Hight: 600 μm  Width: 300 μm  Tip distance: 550 μm | Fracture force: 0.21 N/needle | MRSA infected wounds | Anti- *S. aureus*/ *E. coli* /MRSA;  Anti-bacterial biofilm;  Antioxidant;  Promote angiogenesis | (Yang et al., 2022c) |
| Ag NP | PVA | Polydopamine (PDA) | 12×12 matrix  Shape: pyramid  Hight: 680 μm  Width: 380 μm | Mechanical strength: 0.180 ± 0.014 N/needle  In vivo penetration depth: 114.2-169.7 μm. | *E. coli* infected wounds | Anti- *E. coli* | (Chen et al., 2023a) |
| Ag microparticles | PVA+PVP | Green Tea Extract | 16×16 matrix  Shape: conical  Hight: 850 μm  Width: 300 μm | In vitro insertion depth: 378 μm | *S. aureus* and *P. aeruginosa* infected wounds | Anti- *S. aureus*/ *P. aeruginosa*;  Anti-bacterial biofilm | (Permana et al., 2021) |
| Nanosilver | PEGDA | Sericin | 9×9 matrix  Shape: pyramid tip + rectangular body  Hight: 900 μm  Width: 330 μm | Mechanical strength: 0.4 N/needle;  In vivo insertion depth: 378 μm | NA | Anti- *S. aureus/ P. aeruginosa/ S. epidermidis* | (Gao et al., 2021b) |
| MgH_2_ | PLGA | None | 10×10 matrix  Shape: pyramid  Hight: 500 μm  Width: 200 μm | NA | Diabetic wounds | Reduce oxidative stress;  Promote angiogenesis;  Induction of cell proliferation | (Wang et al., 2023b) |
| Mg^2+^ | Chitosan hydrogel dressing; CS; PVP | Panax notoginseng saponins (PNS) | 20×20 matrix  Shape: conic | Mechanical strength: >0.25 N/needle;  In vivo: transdermal triangles observed | *S. aureus* infected wounds | Anti- *S. aureus/ E. coli*;  Promotes collagen deposition;  Promotes angiogenesis | (Ning et al., 2022b) |
| Mg-MOF | γ-PGA | GO-Ag | Shape: pyramid  Hight: 500 μm  Width: 200 μm | In vivo: Penetrates mouse dermis | Diabetic wounds | Anti- *S. aureus/ E. coli/ P. aeruginosa* | (Yin et al., 2021) |
| HKUST‐1 | PEGDA | GO-MAP/NO | 10×10 matrix  Shape: conic  Hight: 500 μm  Width: 200 μm | In vivo penetration depth: approximately 240 μm | Diabetic wounds | Promotes angiogenesis;  Reduced inflammation | (Yao et al., 2022) |
| Cu^2+^ | PCL+PVP; polyacrylamide (PAM)+PDA | CaO_2_-HA NPs | Porcupine quill-like multilayer MN;  Single layer:  Shape: conic  Hight: 750 μm  Width: 300 μm | Three-layer mechanical strength: 0.117 N/needle;  Maximum detachment forces: 6.21 N/cm^2^ | Diabetic wounds | Anti- *S. aureus/ E. coli*;  Promotes angiogenesis;  Promotes collagen deposition | (Liu et al., 2023b) |
| CuO_2_ | HA | Porous TiO_2_ | 20×20 matrix  Shape: conic  Hight: 600 μm  Width: 250 μm  Tip distance: 600 μm | In vitro: penetrates the dermis of pig skin  In vivo: penetrates the dermis of mice | MRSA infected wounds | Anti- *P. aeruginosa*/MRSA;  Promotes collagen deposition | (Liang et al., 2023) |
| Cu_2_MoS_4_ | PEGDA | Au NP | 10×10 matrix  Shape: conic  Hight: 600 μm  Width: 200 μm | NA | MRSA infected diabetic wounds | Anti- *E. coli* /MRSA;  Reduces oxidative stress;  Decreases glucose levels | (Shan et al., 2023) |
| Zr-MOF (PCN-224) | HA | Dimethyloxalylglycine/meropenem | Shape: conic  Hight: 850 μm  Width: 400 μm | Mechanical strength: 0.9±0.2 N/needle | *S. aureus* infected wounds | Anti- *E. coli / S. aureus/ P. aeruginosa*;  Promotes collagen deposition;  Promotes angiogenesis | (Zeng et al., 2023) |
| Fe NP | Gelatin + polylysine | TA | Shape: conic | Fracture force >0.2 N/needle | Diabetic wounds | Anti- E. coli / S. aureus;  Promotes angiogenesis | (Wang et al., 2023a) |
| Fe NP | HA | Amine-modified mesoporous silica nanoparticles; glucose oxidase (GOx) | 12×12 matrix  Shape: pyramid  Hight: 650 μm  Width: 280 μm  Tip distance: 750 μm | Mechanical strength: 0.06 N/needle | *S. aureus* infected wounds | Anti- *E. coli / S. aureus* | (Li et al., 2023c) |
| TiO_2_ | HA | NA | Shape: pyramid  Hight: 600 μm  Width: 200 μm | NA | S. aureus infected wounds | AB TiO_2_ possesses the highest anti-*S. aureus* activity | (Ouyang et al., 2023) |
