# Supplementary material for: Metallic elements combine with herbal compounds upload in microneedles to promote wound healing: a review
Source: Front Bioeng Biotechnol. 2023 Nov 3;11:1283771. doi: 10.3389/fbioe.2023.1283771 (PMC10655017; doi:10.3389/fbioe.2023.1283771)
Supplement: Supplementary file 2 [file Table2.docx]

**Supplementary table 2:** Structure and properties of microneedle compositions of different herbal compounds; if durg delivery system is dual system and other compounds cell is NA then MNs tip-base is drug-containing vs. drug-absent.

| Herbal compound | Foundation  (base to tip) | Other compounds | Durg delivery system | Microneedle Characteristics | Mechanical performance | Wound types | Efficiency | Reference |
| --- | --- | --- | --- | --- | --- | --- | --- | --- |
| Curcumin made MOF | γ-PGA | Zn- Curcumin MOF | Single system; Dissolving Microneedle | 10×10 matrix  Shape: pyramid  Hight: 466.23±11.72 μm  Width: 260.38±3.02 μm | In vivo: penetrates the cuticle of mouse back skin | Total skin wounds | Reduces oxidative stress;  Promote angiogenesis | (Yang et al., 2023f) |
| Nano curcumin | Gelatin+HA +sodium alginate; HA | New indocyanine Green | Dual system; Dissolving Microneedle | 20×20 matrix  Shape: pyramid  Hight: 540 μm  Width: 300 μm  Tip distance: 700 μm | Mechanical stress: >1.225×10^-2^ N/needle;  In vivo: penetrate the cuticle of mice skin | Total skin wounds | Reduces lymphocyte and neutrophil accumulation | (Shan et al., 2022) |
| Carvacrol NP | Both layers are made of PVP+PVA | NA | Dual system; Dissolving Microneedle | 19×19 matrix  Shape: conic  Hight: 600 μm  Width: 300 μm  Tip distance: 50 μm | In vitro penetration depth: 378 μm;  10.46% decrease in needle height at 32 N force/array | NA | Anti- *S. aureus/ P. aeruginosa* | (Mir et al., 2020) |
| Protocatechuic aldehyde | Type 1: HA; Type 2: HA+ gelatin | NA | Single system; Dissolving Microneedle | Shape: Pyramid  Hight: 1000 μm  Width: 450 μm | In vivo: penetrates the cuticle of normal skin and HS tissue | Hyperplastic scarring | Decreases angiogenic activity;  Increases apoptosis of PA cells;  Inhibits collagen expression | (Hao et al., 2023) |
| Quercetin | Both layers are made of alginate+ HA+ gelatin | Cu^2+^/Zn^2+^ | Dual system; Dissolving Microneedle | Shape: pyramid  Hight: 600 μm  Width: 300 μm | In vivo penetration depth: about 300 μm | Third-degree burn wound | Promotes hair follicle generation;  Promotes angiogenesis | (Zhang et al., 2023b) |
| Quercetin NP | Gelatin | Gallic acid | Single System; Dissolving Microneedle | 9×9 matrix  Shape: pyramid  Hight: 600 μm  Width: 300 μm | Mechanical strength: >0.03N/needle;  In vitro insertion depth: 420μm | NA | Attenuating fibroblast pro-fibrotic gene expression | (Chen et al., 2022d) |
| Quercetin | BSP | CD-MOF/hypertrophic scar fibroblasts membrane | Single System; Dissolving Microneedle | 10×10 matrix  Shape: quadrangular tower-like shape  Hight: 380 μm  Width: 230 μm  Tip distance: 480 μm | Mechanical hardness values: 0.56 N/needle;  In vivo penetration depth: >300 μm | Hyperplastic scarring | Induction of HS cell apoptosis;  Reduces fibrosis;  Anti-inflammatory | (Wu et al., 2021) |
| Asiaticoside | γ-PGA | MXenes | Single System; Hydrogel Microneedle | 10×10 matrix  Shape: pyramid  Hight: 500 μm  Width: 200 μm | In vitro: penetrates the cuticle of dead pig skin | Diabetic wound | Accelerates collagen deposition;  Accelerates angiogenesis | (Wang et al., 2022b) |
| Luteolin | Both layers are made of HA | Nanomotor/ICG/L-Arg | Dual System; Dissolving Microneedle | Shape: conic  Hight: 600 μm  Width: 300 μm | In vivo: penetrates the cuticle of rat | *S. aureus* infected wounds | Anti- *S. aureus* and biofilm formation;  Promotes collagen deposition | (Chen et al., 2023b) |
| Panax notoginseng saponins | Chitosan hydrogel dressing (CSHD); CS; PVP | Mg^2+^ | Triple system; hydrogel microneedle | 20×20 matrix  Shape: conic | Mechanical strength: >0.25 N/needle;  In vivo: transdermal triangles observed in rat skin | S. aureus infected wounds | Anti- *S. aureus/ E. coli;*  *Promotes* collagen deposition;  Promotes angiogenesis | (Ning et al., 2022b) |
| Asiatic acid | *Premna microphylla* extraction | NA | Single System; Hydrogel Microneedle | 15×15 matrix  Shape: Pyramid  Hight: 600 μm  Width: 300 μm  Tip distance: 500 μm | 3% *Premna microphylla* extraction MN compression stress: >0.08 Mmp;  2%: >0.03 Mmp | Total skin wounds | Anti- *E. coli/ S. aureus*;  Promotes angiogenesis | (Chi et al., 2021) |
| Asiatic acid | Type 1 (hydrogel MN): 15%w/w PVA+ 2%w/w CS  Type 2(dissolving MN): 11%w/w PVP+2%w/w CS | NA | Dual system; Hydrogel Microneedle | Shape: pyramid  Hight: 404 μm (type 1); 372 μm (type 1) | Type 1 MN vertical fracture test: 1.26–1.77 N/needle;  Type 2: 0.43–0.51 N/needle | Total skin wounds | Promote collagen deposition;  Promotes fibroblast migration | (Ryall et al., 2022a) |
| Tanshinone II_A_ | Both layers made of PVP/CS | NA | Dual System; Dissolving Microneedle | 10×10 matrix  Shape: conic  Hight: 500 μm  Width: 250 μm  Tip distance: 1000 μm | In vitro: penetrates the cuticle of dead pig skin | NA | Inhibit HSFs proliferation, migration | (Zhan et al., 2023) |
| Shikonin | HA | NA | Single System; Hydrogel Microneedle | Shape: pyramid  Hight: 1000 μm  Width: 300 μm | Mechanical strength: 0.058 N/needle;  In vitro: penetrates the cuticle of dead pig skin | HS wound | Induction of HSF apoptosis | (Ning et al., 2021) |
| BSP | CS/BSP | TA/Ag | Single System; Dissolving Microneedle | Shape: pyramid  Hight: 600 μm  Width: 300 μm  Tip distance: 550 μm | Fracture force: 0.21 N/needle | MRSA infected wounds | Anti- *S. aureus*/ *E. coli* /MRSA;  Anti-bacterial biofilm;  Antioxidant;  Promote angiogenesis | (Yang et al., 2022c) |
| BSP | BSP | mesoporous polydopamine NPS/triamcinolone acetonide | Single System; Dissolving Microneedle | 15×15 matrix  Shape: conic  Hight: 700 μm  Width: 300 μm  Tip distance: 600 μm | In vivo insertion depth: 418 ± 2 μm | Mouth ulcers | Promotes hGFs migration;  Promotes angiogenesis;  Promotes collagen deposition | (Qu et al., 2023) |
| BSP | BSP | NA | Single System; Dissolving Microneedle | 7×7 matrix  Shape: conic  Hight: 781.9 ± 13.1 μm  Width: 318.2 ± 12.5 μm | Mechanical strength: 0.52 N/needle;  In vivo insertion depth: 170-215μm | NA | Low irritation | (Hu et al., 2018) |
| BSP | BSP; GelMA | Yunnanbaiyao (traditional Chinese medicine); EGF | Dual System; Dissolving Microneedle | 20×20 matrix  Shape: conic  Hight: 750 μm  Width: 210 μm  Tip distance: 650 μm | 30% GelMA MN mechanical strength: >0.07 N/needle;  In vivo: penetrates the cuticle of rat skin | Liver wound and total skin wound | Hemostasis;  Promote angiogenesis;  Promote collagen deposition | (Yang et al., 2023c) |
| BSP | Ethyl cellulose; Carboxymethyl chitosan/BSP | Triamcinolone acetonide; HP-β-CD/ TA/Verapami | Dual system; Dissolving microneedles | Shape: conic  Hight: 800 μm  Width: 400 μm | 10mg of drug-containing MN in vivo sertion depth: 200μm;  Maximum capacity: 1.10 N/needle | HS wound | Reduces collagen fiber volume;  Promotes HSF apoptosis | (Zhang et al., 2022a) |
| Panax notoginseng polysaccharide (PNPS) | HA; PNPS | NA | Dual System; Dissolving Microneedle | Shape: Pyramid  Hight: 700 μm  Width: 400 μm  Tip distance: 380 μm | Mechanical strength: 0.16 N/needle;  In vivo insertion depth: 200μm | NA | Stimulates subcutaneous lymphocyte migration;  Activation of T-cells | (Wang et al., 2021) |
