# Supplementary material for: Metallic elements combine with herbal compounds upload in microneedles to promote wound healing: a review
Source: Front Bioeng Biotechnol. 2023 Nov 3;11:1283771. doi: 10.3389/fbioe.2023.1283771 (PMC10655017; doi:10.3389/fbioe.2023.1283771)
Supplement: Supplementary file 3 [file DataSheet1.docx]

Herbal compounds structure image group. Unlabeled chemical formula originated from http://www.chemspider.com/.

1. Curcumin


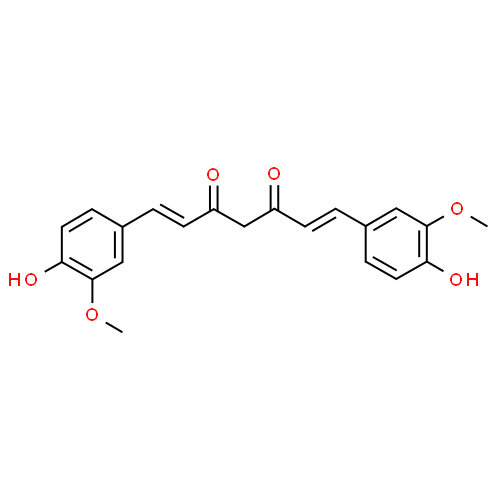


2. Protocatechuic aldehyde


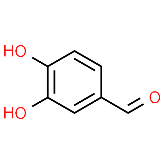


3. Carvacrol


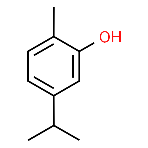


4. Tannic acid


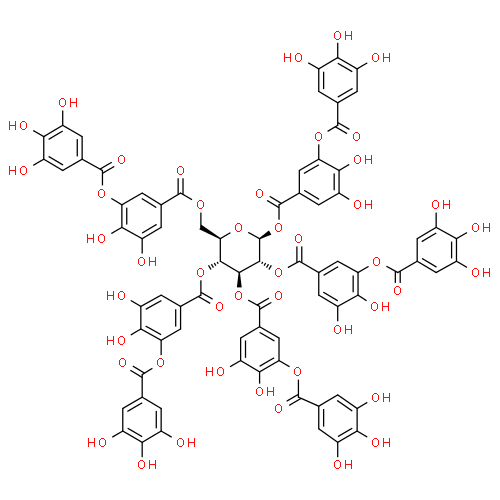


5. Catechin


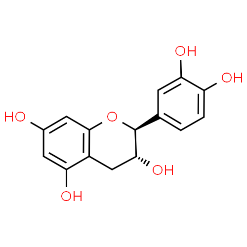


6. Gallic acid


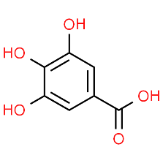


7. Quercetin(Ferenczyova et al., 2020)


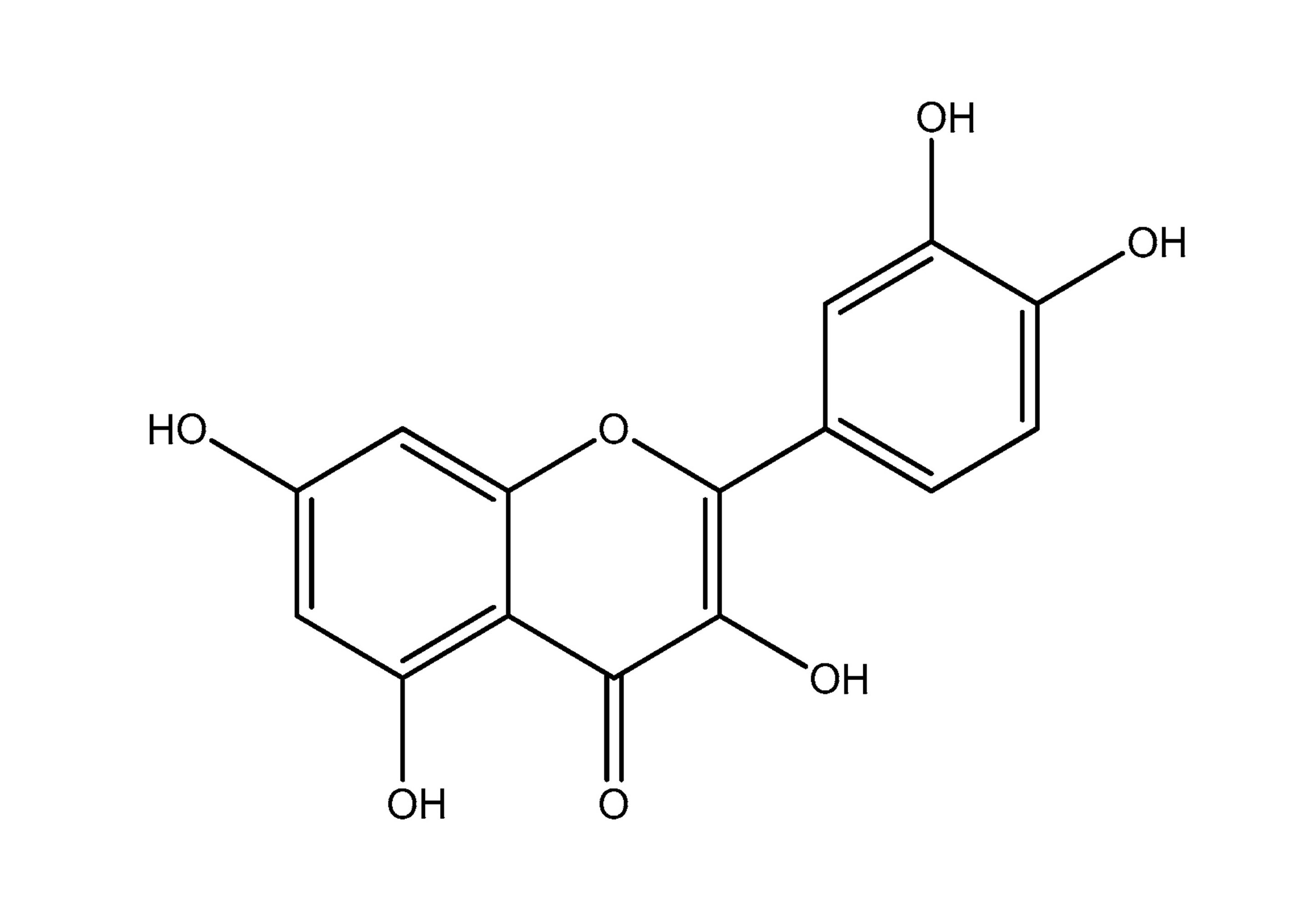


8. Luteolin


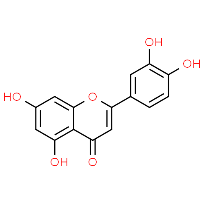


9. Asiatic acid


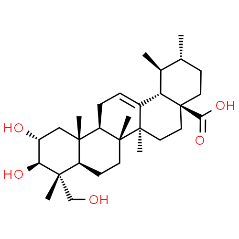


10. Asiaticoside


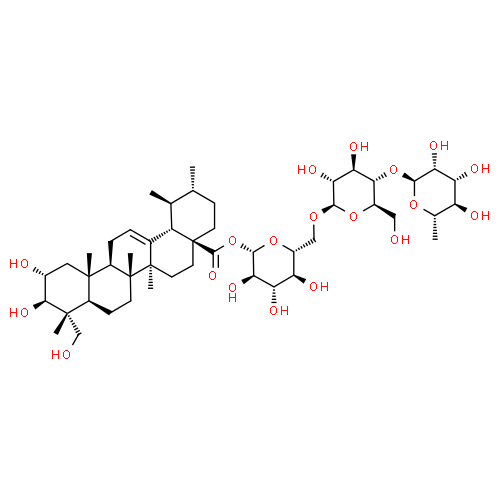


11. Tanshinone II_A_


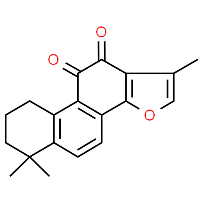


12. Shikonin


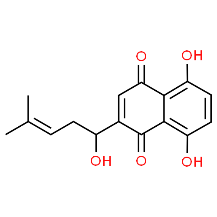


13. Bletilla striata polysaccharide (Qu et al., 2023)


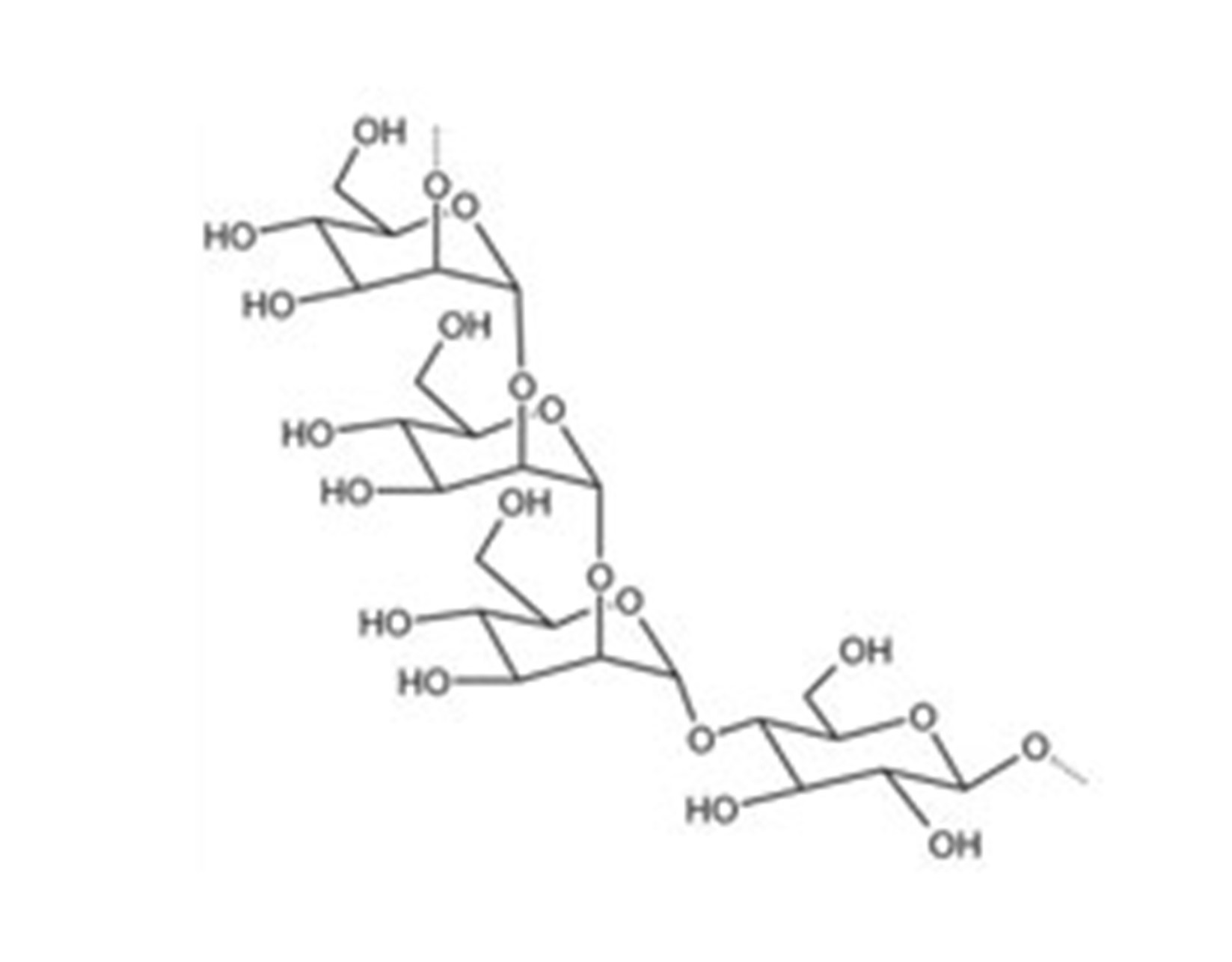


14. Panax notoginseng polysac-charide(Wang et al., 2021)


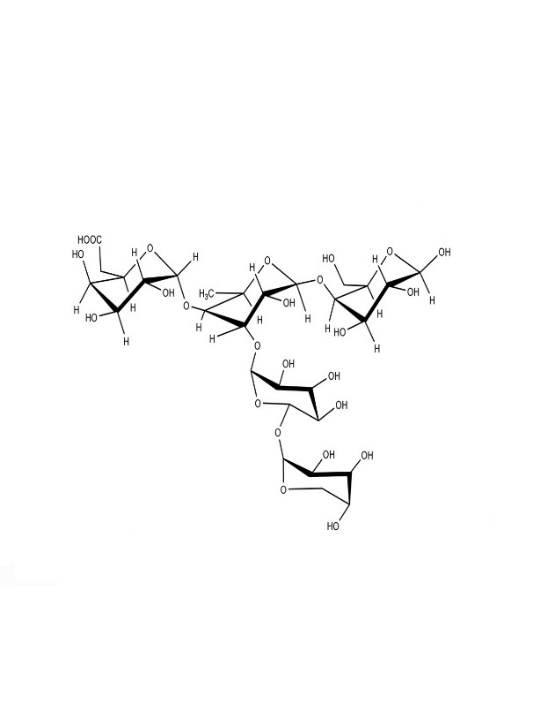


15. Pectin(Chi et al., 2021)


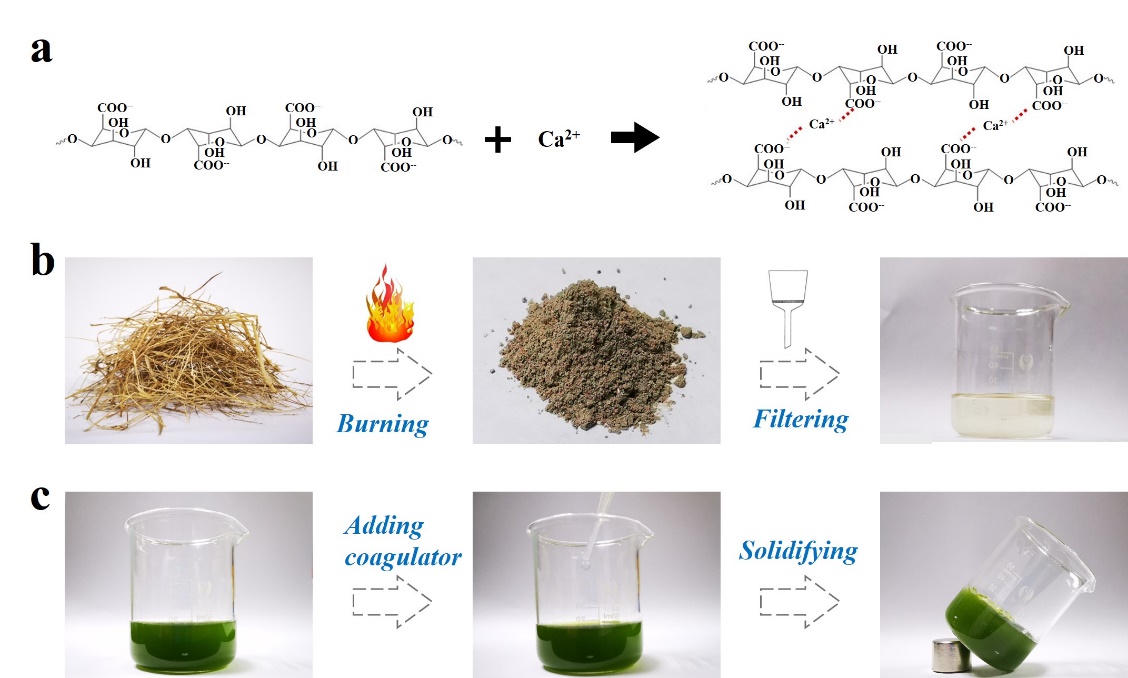


References

Chi, J., Sun, L., Cai, L., Fan, L., Shao, C., Shang, L., et al. (2021). Chinese herb microneedle patch for wound healing. *Bioact Mater* 6(10)**,** 3507-3514. doi: 10.1016/j.bioactmat.2021.03.023.

Ferenczyova, K., Kalocayova, B., and Bartekova, M. (2020). Potential Implications of Quercetin and its Derivatives in Cardioprotection. *Int J Mol Sci* 21(5). doi: 10.3390/ijms21051585.

Qu, X., Guo, X., Zhu, T., Zhang, Z., Wang, W., and Hao, Y. (2023). Microneedle patches containing mesoporous polydopamine nanoparticles loaded with triamcinolone acetonide for the treatment of oral mucositis. *Front Bioeng Biotechnol* 11**,** 1203709. doi: 10.3389/fbioe.2023.1203709.

Wang, C., Liu, S., Xu, J., Gao, M., Qu, Y., Liu, Y., et al. (2021). Dissolvable microneedles based on Panax notoginseng polysaccharide for transdermal drug delivery and skin dendritic cell activation. *Carbohydr Polym* 268**,** 118211. doi: 10.1016/j.carbpol.2021.118211.
